# Magnetic and Electronic Structural Properties of the S_3_ State of Nature’s Water Oxidizing Complex: A Combined Study in ELDOR-Detected Nuclear Magnetic Resonance Spectral Simulation and Broken-Symmetry Density Functional Theory

**DOI:** 10.1021/acsomega.2c06151

**Published:** 2022-11-03

**Authors:** Ciarán J. Rogers, Olivia Hardwick, Thomas A. Corry, Felix Rummel, David Collison, Alice M. Bowen, Patrick J. O’Malley

**Affiliations:** Department of Chemistry and Photon Science Institute, School of Natural Sciences, The University of Manchester, Manchester M13 9PL, U.K.

## Abstract

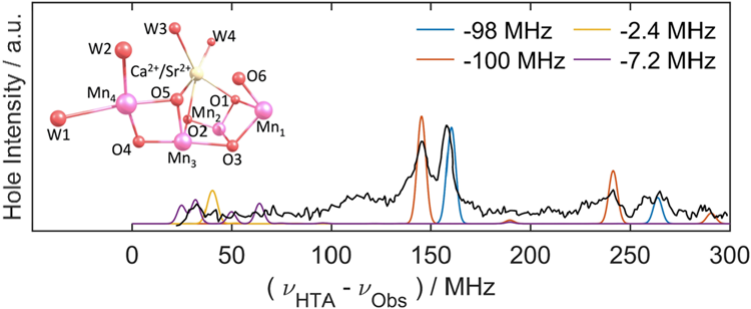

ELDOR-detected nuclear magnetic resonance (EDNMR) spectral simulations combined with broken-symmetry density functional theory (BS-DFT) calculations are used to obtain and to assign the ^55^Mn hyperfine coupling constants (hfcs) for modified forms of the water oxidizing complex in the penultimate S_3_ state of the water oxidation cycle. The study shows that an open cubane form of the core Mn_4_CaO_6_ cluster explains the magnetic properties of the dominant *S* = 3 species in all cases studied experimentally with no need to invoke a closed cubane intermediate possessing a distorted pentacoordinate Mn_4_ ion as recently suggested. EDNMR simulations found that both the experimental bandwidth and multinuclear transitions may alter relative EDNMR peak intensities, potentially leading to incorrect assignment of hfcs. The implications of these findings for the water oxidation mechanism are discussed.

## Introduction

**Figure 1:**
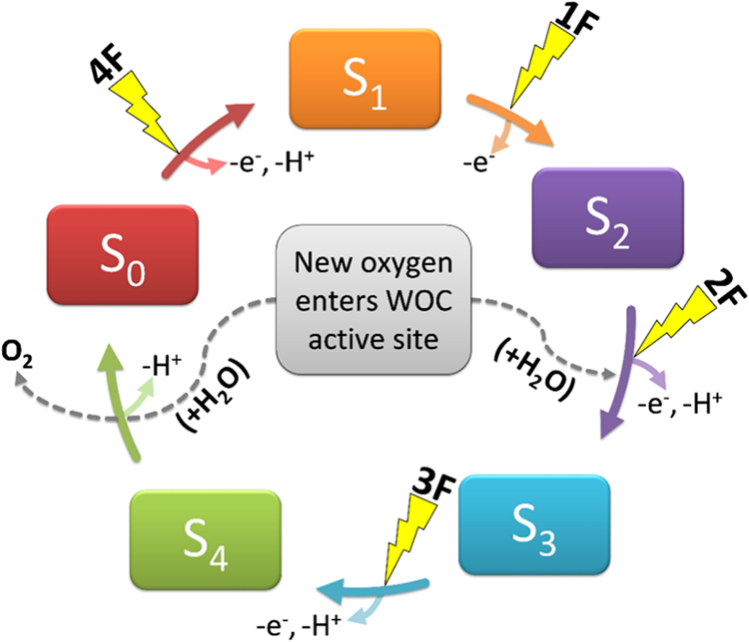
Water oxidizing complex (WOC) S state cycle. The four Mn ions of the complex are progressively oxidized with each photon flash (1F, 2F, 3F, 4F).

**Figure 2:**
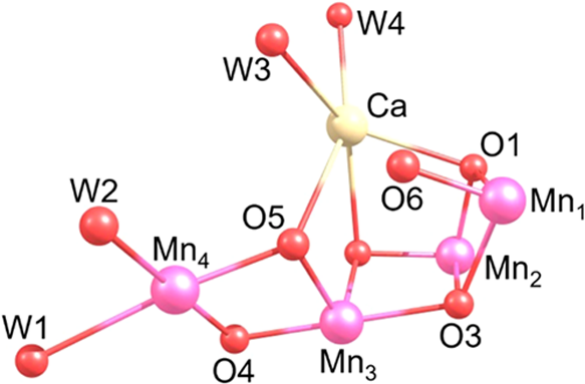
Numbering scheme and orientation used throughout for the S_3_ state core WOC. Color coding: Mn (purple), oxygen (red), and calcium (cream).

The oxidation of two water molecules to form one molecule of oxygen has been performed uniquely at ambient conditions by the water oxidizing complex (WOC) of photosystem II (PSII) for around 3 billion years.^1,2^ Visible light energy is used to sequentially extract four electrons from two water molecules via its core catalytic center, a Mn_4_O_5/6_Ca cluster (Figures 1 and 2). The stepwise S state cycle, Figure 1, allows the WOC to produce oxidized Mn centers, and deprotonated substrate water molecules facilitating the ultimate formation of an O–O bond at the S_3_ or S_4_ state.^3^

Understanding the WOC’s mechanism of low-energy oxidation of water to molecular oxygen has been the subject of a wide number of spectroscopic, crystallographic, and computational studies.^4^ The penultimate S_3_ state is a major current focus of contemporary research as it is the last semistable intermediate before oxygen evolution and has been associated with the oxygen–oxygen bond formation step.^1,5^ The characterization of this state was greatly enhanced by the recent report of an atomic-level X-ray crystal structure of the WOC using X-ray free electron lasers (XFEL), after two light flashes (2F) and poised predominantly in the S_3_ state.^6,7^ The core structure obtained from these studies and the main atom numbering is shown in Figure 2.

High-frequency continuous wave electron paramagnetic resonance (EPR) and ELDOR-detected nuclear magnetic resonance (EDNMR) spectroscopy^8,9^ studies have shown that the S_3_ state gives rise to an *S* = 3 EPR spectrum for the cyanobacterium *Thermosynechococcus elongatus*. The analysis indicated that the four Mn ions are present in the IV oxidation state and octahedrally coordinated. This was supported by broken-symmetry density functional theory (BS-DFT) and Heisenberg–Dirac–van Vleck (HDvV) spin ladder calculations.^10−12^ XFEL crystallographic studies have shown that an O6 atom is detected in the 2F structure in very close proximity to O5. The presence of an oxo–oxyl bond, [O_2_]^3–^ or an equilibrium of these forms has been proposed to describe this.^13−16^ Recently, a five-coordinate precursor S_3_ state was suggested to explain broadened EPR signals in modified forms of the 2F state relative to the native form.^17^ This five-coordinate intermediate, remaining an *S* = 3 system, was proposed to feature a closed cubane (hexacoordinated Mn_1_ and pentacoordinated Mn_4_) rather than an open cubane form (both Mn_1_ and Mn_4_ hexacoordinated) reported previously for the native system, supporting a pivot or carousel mechanism of water oxidation, where Mn_4_ is the binding site for a new water or hydroxide in the S_2_-to-S_3_ transition.^18,19^ Similarly broadened EPR signals have been reported by Marchiori et al.^20^ on glycerol-treated samples, attributing these, by contrast, to an open cubane form. While a closed cubane model has been proposed by many workers for the high-spin S_2_ state and its role in the transition to the S_3_ state, other reports based either on DFT-calculated energetics^21^ or magnetic parameters^22^ have questioned whether such a conformer exists. In one model proposed by Siegbahn,^21^ an extra hydroxo ligand is already bound to Mn_1_ in the S_2_ state, which was proposed to facilitate the formation of the S_3_ state and ruled out the participation of a closed cubane form. In another model, protonation of the O4 μ-oxo was proposed^22^ to give rise to the high-spin form observed by EPR. More recently, it was proposed, based on BS-DFT calculations of hyperfine coupling constants (hfcs) and zero-field splitting (*D*) values, that an open cubane architecture was preferred for both native and alcohol-modified forms.^16^

Assignment of spectral bands from EDNMR data to individual hfcs relies on accurate spin Hamiltonian simulations of both the relative intensity and position of spectral peaks in experimental EDNMR spectra. At high frequencies, EDNMR has become the technique of choice for detecting nuclear transitions in the WOC.^23−25^ This is achieved by employing a long, low-powered high-turning-angle (HTA) microwave pulse to drive polarization transfer between formally forbidden electron transitions (Δ*m*_S_ = ±1; Δ*m*_I_ = ±1), which is subsequently detected as a change in the echo intensity of a simple Hahn Echo or Free Induction Decay (FID) detection sequence. EDNMR can be advantageous over analogous hyperfine techniques, namely, electron nuclear double resonance (ENDOR) spectroscopy, as neither radiofrequency (RF) amplifier bandwidth limitations nor microwave pulse selectivity poses a problem in data collection or analysis, as is the case in Davies ENDOR.^26^ Moreover, EDNMR is robust against fast longitudinal relaxation (*T*_1_).^27^ Previously, EDNMR spectral simulations of the WOC have been performed by approximating the experimental EDNMR spectrum as an ENDOR spectrum using the *salt* routine in EasySpin,^28^ although accurate EDNMR spectral simulations on other systems have been reported based on the Liouville equation.^29^ In this report, we take advantage of the recently reported routine *horseradish*, implemented in EasySpin^30^ and described in detail elsewhere,^31^ but in summary, the *horseradish* simulation considers all allowed and forbidden transitions for a given spin system and calculates the detection probability and inversion efficiency of each center by identifying connected pump transitions, i.e., those related to the frequency of the HTA pulse, giving a more accurate assignment of the spectrally observed EDNMR bands. Importantly, the central blind spot of the EDNMR experiment, where the HTA pulse saturates the detected EPR transition, centered around υ_HTA_ – υ_Obs_ = 0 MHz, is not modeled explicitly in these calculations.

In this report, we combine this simulation method with broken-symmetry density functional theory (BS-DFT) calculations on large models of the WOC (see the Supporting Information, Figure S1). The models are generated from the 2F structure coordinates to calculate the ^55^Mn and ^14^N hfcs to guide our spectral simulations and assignments to specific nuclear peak positions. Our analysis shows that an open cubane structure is appropriate for all modified S_3_ state forms. As previously demonstrated, proposed alcohol binding sites near the O4 position cause small changes in the exchange coupling constants *J* (mainly *J*_34_),^16^ which modifies the projection coefficient for Mn_3_ and Mn_4_ resulting in small-magnitude, negative and isotropic ^55^Mn hfcs (Mn_3–4_). Our EDNMR simulations show that these small-magnitude negative ^55^Mn hfcs are buried in or near the central blind spot for the W-band EDNMR spectra and their relative intensities will have a strong dependence on the assumptions made to correct for the experimental bandwidth of the resonator.

## Results and Discussion

**Figure 3:**
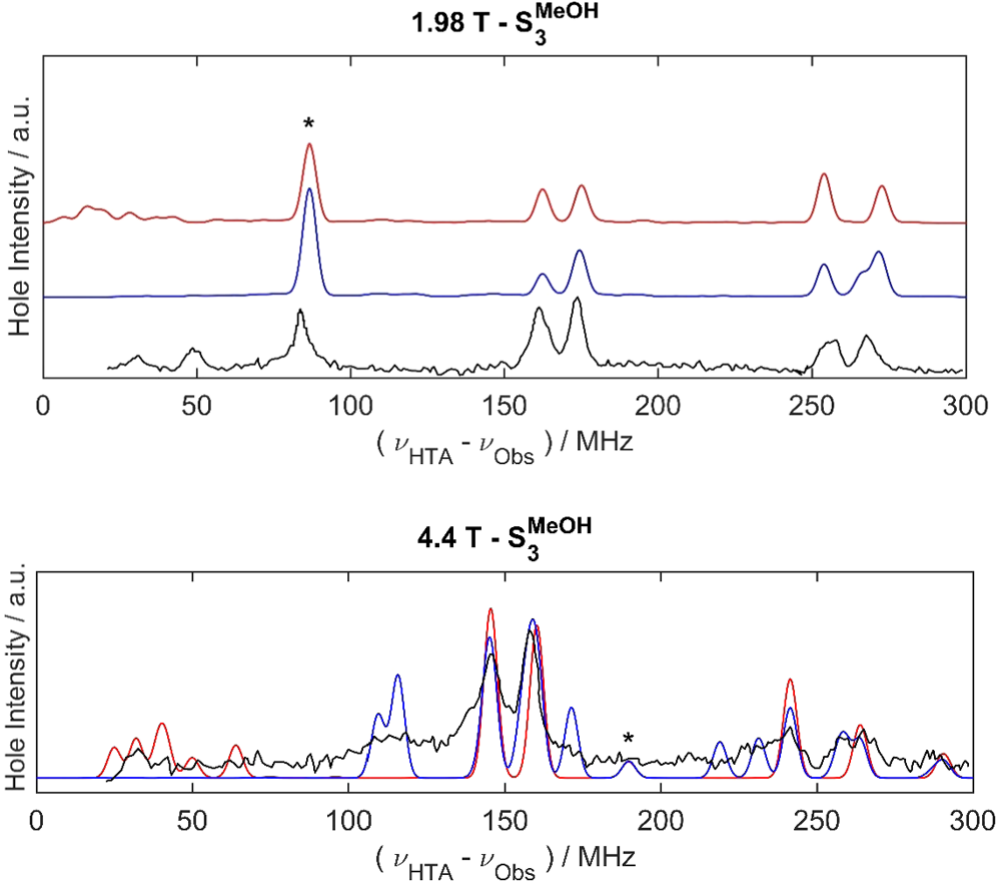
Experimental EDNMR spectra of the methanol-treated PSII reproduced from ref (17) (black); simulated EDNMR spectra summed from simulations with a single ^55^Mn nucleus using the hfcs from Table 2 for closed cubane (blue) and open cubane (red) structures. ^1^H resonances are denoted with asterisks and occur around the proton Larmor frequency. The bottom comparison illustrates spectral overlay for 4.4 T simulation to emphasize fit. All simulated data have been multiplied by a Lorentzian function with an FWHM of 400 MHz to mimic the resonator background.

**Figure 4:**
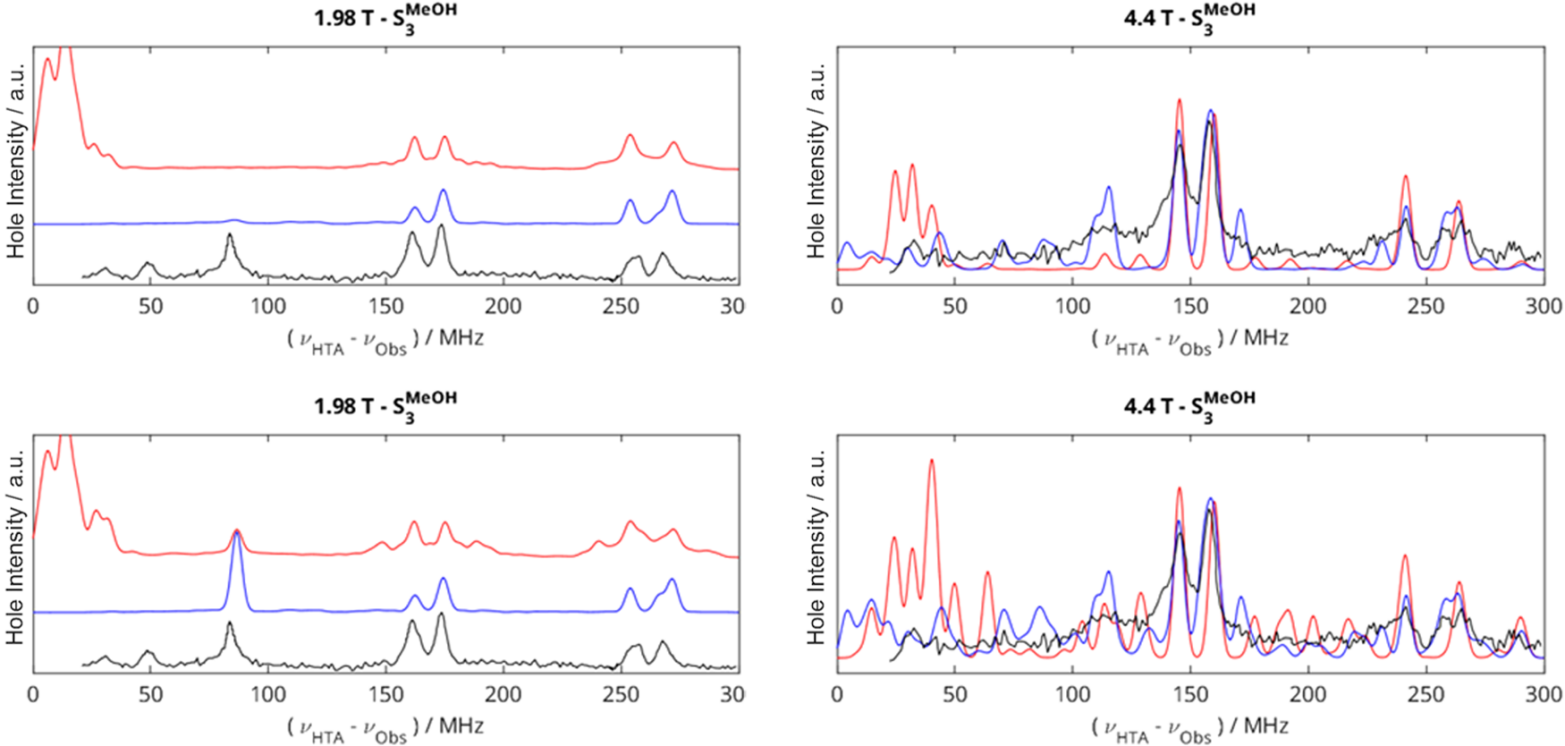
Experimental EDNMR spectra of the methanol-treated PSII reproduced from ref (17) (black); simulated EDNMR spectra summed from simulations with pairs of ^55^Mn nuclei using the hfcs from Table 2 in the main paper for closed cubane (blue) and open cubane (red) structures, calculated at both low and high field positions with an HTA pulse amplitude of 4.7 × 10^6^ (top) and 4.7 × 10^7^ (bottom) rad s^–1^. ^1^H resonances occur around the proton Larmor frequency as shown in Figure 3; 4.4 T simulations are overlaid to emphasize fit. All simulated data have been multiplied by a Lorentzian function with an FWHM of 400 MHz to mimic the resonator background.

Table 1 shows the calculated isotropic and anisotropic ^55^Mn and ^14^N hfcs using BS-DFT calculations on models of methanol and glycerol interactions with the WOC for the S_3_ state. Small-magnitude negative ^55^Mn hfcs are calculated for Mn_3_ and Mn_4_ and large-magnitude negative values, near 100 MHz, are calculated for the Mn_1_ and Mn_2_ positions. The small-magnitude hfcs for the Mn_3_ and Mn_4_ positions can be explained by a dimer of dimers model for the *S* = 3 spin state,^9,10^ or more exactly by the almost equal contribution of the two lowest-energy broken-symmetry states (notated for Mn_1_–Mn_4_) ααβα and αααβ as explained in detail previously.^16^Table 1 also compares the calculated values with those reported by Marchiori et al.^20^ and Chrysina et al.,^17^ who reported open cubane and closed cubane structures, respectively. It is clear that good general agreement is observed between the calculated values and those proposed by Marchiori et al.^20^ Our DFT calculations suggest that the small-magnitude Mn_3_ and Mn_4_ negative hfcs should be interchanged from the Marchiori et al.^20^ assignments. In addition, Marchiori et al.^20^ propose an anisotropic hfc for Mn_4_. This was used to explain the apparently larger zero-field splitting parameter (*D* value) observed for the glycerol-treated form and suggests a distorted octahedral geometry for Mn_4_. However, our calculated values in Table 1 indicate that all of the octahedral Mn(IV) ions give rise to an isotropic ^55^Mn hfc. While it has been assumed that octahedral Mn(IV) ions have low *D* values ≤ 0.3 cm^–1^, this has been shown on both experimental and computational grounds to be incorrect, and distortion of one of the Mn ions is not required to increase the *D* value.^16^ The BS-DFT calculations shown herein indicate that the hfcs of all Mn ions of the complex exhibit low anisotropy. The hfcs reported by Chrysina et al.^17^ and Marchiori et al.^20^ were obtained from simulations of their EDNMR spectra using the salt routine which simulates ENDOR spectra. Implementing the EDNMR-specific *horseradish* routine, we now use the two hyperfine coupling parameter sets, open cubane and closed cubane, in Table 2, to simulate the exact EDNMR spectra at 94 GHz (Figure 3). The EDNMR spectra are simulated at the low, 1.98 T, and high, 4.4 T, field positions of the W-band EPR spectrum to selectively observe only the single-quantum EPR transition, *m*_s_ = |−3> → |−2>, of the methanol-treated species as described previously.^17^ Simulations were performed individually for coupling to each ^55^Mn nucleus and a single proton and then summed^32,33^ (Figure 3), following an approach previously used in the analysis of ENDOR data with couplings to multiple nuclei, and also with each pair of ^55^Mn nuclei and a single proton (Figure 4). In each case, a Lorentzian function with an FWHM of 400 MHz was applied to correct for the inhomogeneities of the B_1_ field across the bandwidth of the resonator (see the SI; Bandwidth considerations in simulating EDNMR spectra). For the simulations in which there was coupling to two or more ^55^Mn nuclei, deviations from the summation of the individual simulations were observed at high powers of the HTA microwave pulse, due to multi-quantum transitions involving two or more nuclei. These multi-quantum transitions may be responsible for some of the signals in the experimental EDNMR traces.

**Table 1:** Calculated Isotropic (*A*_iso_) and Anisotropic (*T*_nn_) ^55^Mn and ^14^N hfcs by BS-DFT for the S_3_ Model Compared with Experimental Values Determined from Simulations^a^

atom labeling from Figure 2		calculated by DFT in this study	Marchiori et al.^20^	Chrysina et al.^17^
Mn_4_	*A*_iso_	–7.2	–1.7	62.3
	*T*_11_	0	–1.3	64.7
	*T*_22_	0	0.7	–30.3
	*T*_33_	0	0.7	–34.3
Mn_3_	*A*_iso_	–2.4	–7.5	–94.8
	*T*_11_	0	0	3.5
	*T*_22_	0	0	–1.7
	*T*_33_	0	0	–1.7
Mn_2_	*A*_iso_	–100	–96	–98.8
	*T*_11_	–6	–5	–3.6
	*T*_22_	2	1	1.8
	*T*_33_	4	4	1.8
Mn_1_	*A*_iso_	–98	–99	–101.8
	*T*_11_	–6	–7	–1.2
	*T*_22_	1	2	–1.2
	*T*_33_	5	5	–4.3
^14^N (D1-His332)	*A*_iso_	–1.3	ND	ND

a All values are given in MHz.

**Table 2:** Hyperfine Couplings (MHz) Used for *Horseradish* EDNMR Spectral Simulations Presented in Figure 3

W-band (94 GHz)	hyperfine values (MHz) used in simulations				
hyperfine matrix	Mn1	Mn2	Mn3	Mn4	^1^H
[*A*_11_, *A*_22_, *A*_33_]—this work (open cubane)	[−104, −104, −97.5]	[−96.5, −96.5, −91.3]	–2.4	–7.2	1
[*A*_11_, *A*_22_, *A*_33_]—Chrysina et al.^17^ (closed cubane)	[−104, −104, −97.5]	[−97, −102.5, −97]	[−96.5, −96.5, −91.3]	[28, 32, 127]	1

It is clear by the inspection of Figure 3 that the simulation of the closed cubane hfcs by Chrysina et al.^17^ differs from the observed EDNMR spectra, particularly at the high field position of 4.4 T, where sharp signals are calculated at ca. 105, 115, and 170 MHz that are not seen to the same extent in the experimental data. At the low field position of 1.98 T, the closed cubane set provides a reasonable fit to the experimental data; however, the weaker spectral bands at and below 50 MHz—assigned to the anisotropic Mn_4_ of the closed cubane representation—are not present in the *horseradish* simulation using these values. The two large-magnitude hfcs (^55^Mn_1_ and ^55^Mn_2_) are very well reproduced at both low and high field positions and are in agreement with the values of both Chrysina et al.^17^ and Marchiori et al.^20^

For the small-magnitude pair of hfcs (^55^Mn_3_ and ^55^Mn_4_), two prominent single-quantum EDNMR transition frequencies are expected for each coupling, given by υ_Mn_^55^ ± 3|*A*| and υ_Mn_^55^ ± 2|*A*|, with + and – corresponding to positive and negative hfc values, respectively.^20^ The small negative values of the hfcs as calculated by our BS-DFT calculations, Table 1, result in the simulation of spectral peaks of low intensity that lie in a region of the spectrum overlapping and obscured by the central blind spot of the EDNMR experiment. The shape of the central blind spot and experimental background will be greatly affected by B_1_ inhomogeneities. Consequently, post-processing approximations may distort the experimental data, particularly in the low-frequency region.^25^ The inhomogeneity of the B_1_ field at different offset frequencies will further affect the experimental background and relative intensities of the spectral peaks at different frequencies, particularly around the maximum of the B_1_ field, where changes in B_1_ are largest with respect to changes in offset frequency. Such uncertainties may explain the non-observance of the EDNMR bands below ∼60 MHz in the W-band (94 GHz) spectra for the methanol-treated samples, as the relative ratio of these peaks to the more well-defined signals at ca. 150 MHz depends on the bandwidth of the resonator.

The simulations presented in Figure 3 do not consider multi-quantum transitions (MQTs) involving more than one ^55^Mn nucleus. Results from simulations including each pair of ^55^Mn nuclei show that for the simulation at 4.4 T using the hfcs for the open cubane structure (Table 2) when some MQTs are included, new signals are observed between 100 and 140 MHz, indicating that the broad experimental signal observed at 120 MHz may be due to MQTs. Using the hfcs for both the open and closed cubane cases (Table 2), the inclusion of MQTs arising from two ^55^Mn nuclei leads to more peaks in the EDNMR spectrum at all frequencies that are not resolved in the experimental data. In particular, both sets of hfcs lead to more MQTs at lower-frequency offsets in the 4.4 T simulation. The number and amplitude of these MQT peaks are reduced in a simulation carried out with an HTA of lower power. This highlights the importance of knowing the bandwidth of the resonator to accurately model EDNMR simulations, particularly in cases where MQTs may be important (further details in the SI, Consideration of multi-quantum effects in EDNMR).

It is important to note that linewidth parameters are added to provide best agreement with the experimental data in all sets of simulations. Experimentally, the observed linewidth depends on the experimental parameters including the pulse lengths used in the detection sequence. Here, the linewidth broadening of simulated peaks is given as a Gaussian with an FWHM of 5 MHz, which reproduces well all major experimental peaks. The linewidth parameter also encodes for the strain of hyperfine values, representing a variation across the molecular configurations present in the sample. It is possible that such variations in the open cubane structure provide larger strains in the hfcs of Mn_3_ and Mn_4_, thus reducing the observed amplitude of EDNMR signals from these couplings relative to Mn_1_ and Mn_2_. In the simulations of Chrysina et al.*,*^17^ using the ENDOR algorithm, a linewidth for the anisotropic Mn_4_ coupling 3 times larger than that for any other coupling was used.^17^

Further supporting the open cubane model, the glycerol-treated samples studied by Marchiori et al.^20^ show a similarly elevated *D* value to the methanol-treated system, indicating that a similar perturbation occurs, and further, using the *horseradish* algorithm to re-simulate the experimental data replicates well all experimental peaks (see the SI). In this study, higher-frequency measurements at 130 GHz (D-band), compared to 94 GHz (W-band) by Chrysina et al.*,*^17^ further increase the ^55^Mn Larmor frequency, pushing the EDNMR transitions to higher-frequency values and away from the central blind spot. However, at higher fields, an overlap of small hyperfine values with a ^14^N spectral peak (υ_N_^14^ = 17.5 MHz at 5.7 T) begins to occur. Two-dimensional (2D)-EDNMR spectroscopy may help to further aid in signal assignment of the overlapping signals of small-magnitude transitions and ^14^N transitions at higher fields.^29^

## Conclusions

Broken-symmetry density functional theory (BS-DFT) calculations combined with ELDOR-detected nuclear magnetic resonance (EDNMR) spectral simulations have been used to assign the ^55^Mn hfcs for modified spectral forms of the water oxidizing complex (WOC) in the penultimate S_3_ state of the water oxidation cycle. The analysis indicates that an open cubane form of an all-octahedral Mn ion, oxo (O5)–hydroxo (O6), WOC cluster is the dominant *S* = 3 species observed in all perturbed cases studied experimentally with no need to invoke a closed cubane intermediate possessing a distorted pentacoordinate Mn ion. Currently popular mechanisms for water oxidation, invoking a closed cubane form of the WOC complex as an intermediate state, are not supported by this study.

### Methodology and Computational Details

The computational procedure is similar to that described previously in detail.^12,22,34^ Full details are given in the SI.

## Supporting Information Available

Full details of computational and simulation methods used; illustrated and *XYZ* coordinates of models used in BS-DFT calculations; supporting EDNMR simulations; bandwidth considerations in simulating EDNMR spectra; and consideration of multi-quantum effects in EDNMR (PDF)

## References

[ref1] Renger G. Mechanism of light induced water splitting in Photosystem II of oxygen evolving photosynthetic organisms. Biochim. Biophys. Acta, Bioenerg. 2012, 1817, 1164–1176. 10.1016/j.bbabio.2012.02.005. 22353626

[ref2] Shevela D.; Kern J. F.; Berkeley L.; Whitmarsh J.; Messinger J.; Dennis P. Photosystem II. Biochem. Soc. Trans. 2001, 6, 901–913.

[ref3] Kok B.; Forbush B.; McGloin M. Cooperation of Charges In Photosynthetic O_2_ Evolution–I. A Linear Four Step Mechanism. Photochem. Photobiol. 1970, 11, 457–475. 10.1111/j.1751-1097.1970.tb06017.x. 5456273

[ref4] Pantazis D. A. Missing Pieces in the Puzzle of Biological Water Oxidation. ACS Catal. 2018, 8, 9477–9507. 10.1021/acscatal.8b01928.

[ref5] Corry T. A.; O’Malley P. J. Electronic-Level View of O-O Bond Formation in Nature’s Water Oxidizing Complex. J. Phys. Chem. Lett. 2020, 11, 4221–4225. 10.1021/acs.jpclett.0c00794. 32374174

[ref6] Suga M.; Akita F.; Sugahara M.; Kubo M.; Nakajima Y.; Nakane T.; Yamashita K.; Umena Y.; Nakabayashi M.; Yamane T.; Nakano T.; Suzuki M.; Masuda T.; Inoue S.; Kimura T.; Nomura T.; Yonekura S.; Yu L.-J.; Sakamoto T.; Motomura T.; Chen J.-H.; Kato Y.; Noguchi T.; Tono K.; Joti Y.; Kameshima T.; Hatsui T.; Nango E.; Tanaka R.; Naitow H.; Matsuura Y.; Yamashita A.; Yamamoto M.; Nureki O.; Yabashi M.; Ishikawa T.; Iwata S.; Shen J.-R. Light-induced structural changes and the site of O=O bond formation in PSII caught by XFEL. Nature 2017, 543, 131–135. 10.1038/nature21400. 28219079

[ref7] Young I. D.; Ibrahim M.; Chatterjee R.; Gul S.; Fuller F. D.; Koroidov S.; Brewster A. S.; Tran R.; Alonso-Mori R.; Kroll T.; Michels-Clark T.; Laksmono H.; Sierra R. G.; Stan C. A.; Hussein R.; Zhang M.; Douthit L.; Kubin M.; de Lichtenberg C.; Vo Pham L.; Nilsson H.; Cheah M. H.; Shevela D.; Saracini C.; Bean M. A.; Seuffert I.; Sokaras D.; Weng T.-C.; Pastor E.; Weninger C.; Fransson T.; Lassalle L.; Bräuer P.; Aller P.; Docker P. T.; Andi B.; Orville A. M.; Glownia J. M.; Nelson S.; Sikorski M.; Zhu D.; Hunter M. S.; Lane T. J.; Aquila A.; Koglin J. E.; Robinson J.; Liang M.; Boutet S.; Lyubimov A. Y.; Uervirojnangkoorn M.; Moriarty N. W.; Liebschner D.; Afonine P. V.; Waterman D. G.; Evans G.; Wernet P.; Dobbek H.; Weis W. I.; Brunger A. T.; Zwart P. H.; Adams P. D.; Zouni A.; Messinger J.; Bergmann U.; Sauter N. K.; Kern J.; Yachandra V. K.; Yano J. Structure of photosystem II and substrate binding at room temperature. Nature 2016, 540, 453–457. 10.1038/nature20161. 27871088 PMC5201176

[ref8] Boussac A.; Sugiura M.; Rutherford A. W.; Dorlet P. Complete EPR spectrum of the S_3_-state of the oxygen-evolving photosystem II. J. Am. Chem. Soc. 2009, 131, 5050–5051. 10.1021/ja900680t. 19320479

[ref9] Cox N.; Retegan M.; Neese F.; Pantazis D. A.; Boussac A.; Lubitz W. Electronic structure of the oxygen-evolving complex in photosystem II prior to O-O bond formation. Science 2014, 345, 804–808. 10.1126/science.1254910. 25124437

[ref10] Krewald V.; Retegan M.; Cox N.; Messinger J.; Lubitz W.; DeBeer S.; Neese F.; Pantazis D. A. Metal oxidation states in biological water splitting. Chem. Sci. 2015, 6, 1676–1695. 10.1039/C4SC03720K. 29308133 PMC5639794

[ref11] Beal N. J.; Corry T. A.; O’Malley P. J. A Comparison of Experimental and Broken Symmetry Density Functional Theory (BS-DFT) Calculated Electron Paramagnetic Resonance (EPR) Parameters for Intermediates Involved in the S_2_ to S_3_ State Transition of Nature’s Oxygen Evolving Complex. J. Phys. Chem. B 2018, 122, 1394–1407. 10.1021/acs.jpcb.7b10843. 29300480

[ref12] Corry T. A.; O’Malley P. J. Evidence of O–O Bond Formation in the Final Metastable S_3_ State of Nature’s Water Oxidizing Complex Implying a Novel Mechanism of Water Oxidation. J. Phys. Chem. Lett. 2018, 9, 6269–6274. 10.1021/acs.jpclett.8b02793. 30336040

[ref13] Suga M.; Akita F.; Yamashita K.; Nakajima Y.; Ueno G.; Li H.; Yamane T.; Hirata K.; Umena Y.; Yonekura S.; Yu L.; Murakami H.; Nomura T.; Kimura T.; Kubo M.; Baba S.; Kumasaka T.; Tono K.; Yabashi M.; Isobe H.; Yamaguchi K.; Yamamoto M.; Ago H.; Shen J. An oxyl/oxo mechanism for oxygen-oxygen coupling in PSII revealed by an X-ray free-electron laser. Science 2019, 366, 334–338. 10.1126/science.aax6998. 31624207

[ref14] Kern J.; Chatterjee R.; Young I. D.; Fuller F. D.; Lassalle L.; Ibrahim M.; Gul S.; Fransson T.; Brewster A. S.; Alonso-Mori R.; Hussein R.; Zhang M.; Douthit L.; de Lichtenberg C.; Cheah M. H.; Shevela D.; Wersig J.; Seuffert I.; Sokaras D.; Pastor E.; Weninger C.; Kroll T.; Sierra R. G.; Aller P.; Butryn A.; Orville A. M.; Liang M.; Batyuk A.; Koglin J. E.; Carbajo S.; Boutet S.; Moriarty N. W.; Holton J. M.; Dobbek H.; Adams P. D.; Bergmann U.; Sauter N. K.; Zouni A.; Messinger J.; Yano J.; Yachandra V. K. Structures of the intermediates of Kok’s photosynthetic water oxidation clock. Nature 2018, 563, 421–425. 10.1038/s41586-018-0681-2. 30405241 PMC6485242

[ref15] Mandal M.; Saito K.; Ishikita H. The Nature of the Short Oxygen–Oxygen Distance in the Mn_4_CaO_6_ Complex of Photosystem II Crystals. J. Phys. Chem. Lett. 2020, 11, 10262–10268. 10.1021/acs.jpclett.0c02868. 33210928

[ref16] Corry T. A.; O’Malley P. J. S_3_ State Models of Nature’s Water Oxidizing Complex: Analysis of Bonding and Magnetic Exchange Pathways, Assessment of Experimental Electron Paramagnetic Resonance Data, and Implications for the Water Oxidation Mechanism. J. Phys. Chem. B 2021, 125, 10097–10107. 10.1021/acs.jpcb.1c04459. 34463499

[ref17] Chrysina M.; Heyno E.; Kutin Y.; Reus M.; Nilsson H.; Nowaczyk M. M.; DeBeer S.; Neese F.; Messinger J.; Lubitz W.; Cox N. Five-coordinate Mn^IV^ intermediate in the activation of nature’s water splitting cofactor. Proc. Natl. Acad. Sci. U.S.A. 2019, 116, 16841–16846. 10.1073/pnas.1817526116. 31391299 PMC6708314

[ref18] Askerka M.; Brudvig G. W.; Batista V. S. The O_2_ -Evolving Complex of Photosystem II: Recent Insights from Quantum Mechanics/Molecular Mechanics (QM/MM), Extended X-ray Absorption Fine Structure (EXAFS), and Femtosecond X-ray Crystallography Data. Acc. Chem. Res. 2017, 50, 41–48. 10.1021/acs.accounts.6b00405. 28001034

[ref19] Retegan M.; Krewald V.; Mamedov F.; Neese F.; Lubitz W.; Cox N.; Pantazis D. A. A five-coordinate Mn(IV) intermediate in biological water oxidation: spectroscopic signature and a pivot mechanism for water binding. Chem. Sci. 2016, 7, 72–84. 10.1039/C5SC03124A. 29861966 PMC5950799

[ref20] Marchiori D. A.; Debus R. J.; Britt R. D. Pulse EPR Spectroscopic Characterization of the S_3_ State of the Oxygen-Evolving Complex of Photosystem II Isolated from *Synechocystis*. Biochemistry 2020, 59, 4864–4872. 10.1021/acs.biochem.0c00880. 33319991

[ref21] Siegbahn P. E. M. The S_2_ to S_3_ transition for water oxidation in PSII (photosystem II), revisited. Phys. Chem. Chem. Phys. 2018, 20, 22926–22931. 10.1039/C8CP03720E. 30152818

[ref22] Corry T. A.; O’Malley P. J. Molecular Identification of a High-Spin Deprotonated Intermediate during the S_2_ to S_3_ Transition of Nature’s Water-Oxidizing Complex. J. Am. Chem. Soc. 2020, 142, 10240–10243. 10.1021/jacs.0c01351. 32431144

[ref23] Navarro M. P.; Ames W. M.; Nilsson H.; Lohmiller T.; Pantazis D. A.; Rapatskiy L.; Nowaczyk M. M.; Neese F.; Boussac A.; Messinger J.; Lubitz W.; Cox N. Ammonia binding to the oxygen-evolving complex of photosystem II identifies the solvent-exchangeable oxygen bridge (μ-oxo) of the manganese tetramer. Proc. Natl. Acad. Sci. U.S.A. 2013, 110, 15561–15566. 10.1073/pnas.1304334110. 24023065 PMC3785721

[ref24] Mino H.; Ono T. Applications of pulsed ELDOR-detected NMR measurements to studies of photosystem II: Magnetic characterization of YD tyrosine radical and Mn^2+^ bound to the high-affinity site. Appl. Magn. Reson. 2003, 23, 571–583. 10.1007/BF03166642.

[ref25] Möbius K.; Lubitz W.; Cox N.; Savitsky A. Biomolecular EPR Meets NMR at High Magnetic Fields. Magnetochemistry 2018, 4, 50 10.3390/magnetochemistry4040050.

[ref26] Davies E. R. A New Pulse ENDOR Technique. Phys. Lett. A 1974, 47, 1–2. 10.1016/0375-9601(74)90078-4.

[ref27] Goldfarb D. Eldor-detected NMR. eMagRes 2017, 6, 101–104. 10.1002/9780470034590.emrstm1516.

[ref28] Cox N.; Nalepa A.; Lubitz W.; Savitsky A. ELDOR-detected NMR: A general and robust method for electron-nuclear hyperfine spectroscopy?. J. Magn. Reson. 2017, 280, 63–78. 10.1016/j.jmr.2017.04.006. 28579103

[ref29] Kaminker I.; Wilson T. D.; Savelieff M. G.; Hovav Y.; Zimmermann H.; Lu Y.; Goldfarb D. Correlating nuclear frequencies by two-dimensional ELDOR-detected NMR spectroscopy. J. Magn. Reson. 2014, 240, 77–89. 10.1016/j.jmr.2013.12.016. 24530956

[ref30] Stoll S.; Schweiger A. EasySpin, a comprehensive software package for spectral simulation and analysis in EPR. J. Magn. Reson. 2006, 178, 42–55. 10.1016/j.jmr.2005.08.013. 16188474

[ref31] Wili N.; Richert S.; Limburg B.; Clarke S. J.; Anderson H. L.; Timmel C. R.; Jeschke G. ELDOR-detected NMR beyond hyperfine couplings: A case study with Cu(II)-porphyrin dimers. Phys. Chem. Chem. Phys. 2019, 21, 11676–11688. 10.1039/C9CP01760G. 31134254

[ref32] Abdalla J. A. B.; Bowen A. M.; Bell S. G.; Wong L. L.; Timmel C. R.; Harmer J. Characterisation of the paramagnetic [2Fe–2S]^+^ centre in palustrisredoxin-B (PuxB) from *Rhodopseudomonas palustris* CGA009: *g*-matrix determination and spin coupling analysis. Phys. Chem. Chem. Phys. 2012, 14, 6526–6537. 10.1039/c2cp24112a. 22460919

[ref33] Lee H. B.; Marchiori D. A.; Chatterjee R.; Oyala P. H.; Yano J.; Britt R. D.; Agapie T. *S* = 3 Ground State for a Tetranuclear Mn_4_^IV^O_4_ Complex Mimicking the S_3_ State of the Oxygen Evolving Complex. J. Am. Chem. Soc. 2020, 142, 3753–3761. 10.1021/jacs.9b10371. 32013412 PMC7236085

[ref34] Corry T. A.; O’Malley P. J. Proton Isomers Rationalize the High- and Low-Spin Forms of the S_2_ State Intermediate in the Water-Oxidizing Reaction of Photosystem II. J. Phys. Chem. Lett. 2019, 10, 5226–5230. 10.1021/acs.jpclett.9b01372. 31429574

