# Supplementary material for: Magnetic and Electronic Structural Properties of the S3 State of Nature’s Water Oxidizing Complex: A Combined Study in ELDOR-Detected Nuclear Magnetic Resonance Spectral Simulation and Broken-Symmetry Density Functional Theory
Source: ACS Omega. 2022 Nov 3;7(45):41783–8. doi: 10.1021/acsomega.2c06151 (PMC9670293; doi:10.1021/acsomega.2c06151)
Supplement: Supplementary file 1 — ao2c06151_si_001.pdf [file ao2c06151_si_001.pdf]

Supporting Information for

The magnetic and electronic structural properties of the S_3 state of Nature's water oxidising complex: a combined study in ELDOR-Detected Nuclear Magnetic Resonance spectral simulation and Broken Symmetry Density Functional Theory.

Ciarán J. Rogers,[a] Olivia Hardwick,[a] Thomas A. Corry,[a] Felix Rummel,[a] David Collison,[a] Alice M. Bowen[a]* and Patrick J. O'Malley[a]*

[a] Department of Chemistry and Photon Science Institute, The University of Manchester, Oxford Road, Manchester M13 9PL, U.K

*Email: alice.bowen@manchester.ac.uk

*Email: patrick.omalley@manchester.ac.uk

Table of contents

Broken Symmetry Density Functional Theory (BS-DFT)..2
XYZ coordinates of methanol model used in BS-DFT calculations.....................................5
XYZ coordinates of glycerol model used in BS-DFT calculations.....................................13

ELDOR-detected NMR (EDNMR) simulations 21
Supplementary EDNMR simulations 22
Bandwidth considerations in simulating EDNMR spectra...24
Consideration of multi-quantum effects in EDNMR 26

S1 Broken Symmetry Density Functional Theory (BS-DFT)

Methods

All calculations were performed using ORCA 4[1] with models initially geometry optimised in the fully ferromagnetic state of $(Mn^{IV})_4$.[2] Optimisation calculations used the BP86 functional[3,4] with the zeroth-order regular approximation (ZORA) Hamiltonian applied to include scalar relativistic effects.[5–7] ZORA versions of the def2-SVP basis sets were used for C and H atoms. ZORA versions of the def2-TZVP basis set with f functions removed were used for all other atoms.[8] The resolution of identity approximation (RI-J) for coulomb integrals, along with the decontracted general Weigend auxiliary basis sets were used.[9–11] Dispersion corrections proposed by Grimme with Becke-Johnson damping (D3BJ) were included.[12,13] The conductor-like polarizable continuum model (CPCM) with a dielectric constant $\varepsilon = 8.0$[14,15] was applied in all calculations. Increased integration grids (Grid6 and IntAcc 6 in ORCA convention) and tight SCF convergence criteria were used. All terminal carbon atoms were constrained during optimisation calculations. Heisenberg exchange coupling constants (J_{ij}) and hyperfine couplings were calculated using the broken symmetry DFT (BS-DFT) methodology proposed and justified by Pantazis *et al.*[14,16] The hybrid meta-GGA TPSSh functional was used for BS-DFT and EPR calculations. The chain of spheres (RIJCOSX) approximation to exact exchange was applied along with the same decontracted auxillary basis sets involved in geometry optimization calculations.[17,18] ZORA versions of the def2-TZVP basis set with f functions removed was used for all atoms.[8] Initial BS guesses were constructed using the 'flipspin' feature of ORCA.[19] Convergence to the correct BS and HS states in all calculations was confirmed by examination of the calculated Mulliken spin populations. The 'orca_eca' module within ORCA was used to calculate HDvV spin ladders and spin projection coefficients. Calculation of the hfcs used basis sets for Mn, N and O atoms based on SARC def2-TZVP which have been developed by Neese et al.[20] These basis sets contained fully decontracted s-shells with three additional steep primitives added to the core, in addition to this the def2-TZVP(-f) basis set was used for all other atoms.[11,20] Increased integration grids were used for Mn, N and O, these were set to 11, 9 and 9, respectively. Picture change effects were applied for the

calculation of hfcs. ^{55}Mn isotropic hfcs were scaled by an empirically derived factor of 1.7 to account for well known deficiencies of the Fermi contact term when calculated using DFT methods.[21,22]

For the S_3 state of the WOC with $Mn_{1\text{-}4}$ oxidation states IV–IV–IV–IV, a local S_z of 3/2 for the d^3 ion Mn(IV) is used. The high-spin determinant can be described as |+3/2, +3/2, +3/2, +3/2> (or "αααα", M_S = 12) and six BS determinants were created by inverting local spins of Mn ions. For example, |+3/2, +3/2, +3/2, -3/2> ("βααα", M_S = 3) or |+3/2,-3/2, -3/2, +3/2> ("αββα", M_S = 0). Each BS determinant is described by an M_S value, rather than S, as this is not a spin eigenfunction. The relative energy of each BS determinant from the HS state was used to calculate a set of J_{ij} values for the S_z-only (Ising) Hamiltonian. The calculated exchange coupling constants are used to diagonalise the full HDvV Hamiltonian. The outcome of this step provides the resultant spin ladder and spin projection coefficients. The full details of the procedure have been described extensively in the literature and has been used successfully for many exchange-coupled Mn systems.[14,16,23]

Models used are described in the main text and more extensively characterised in reference [24]

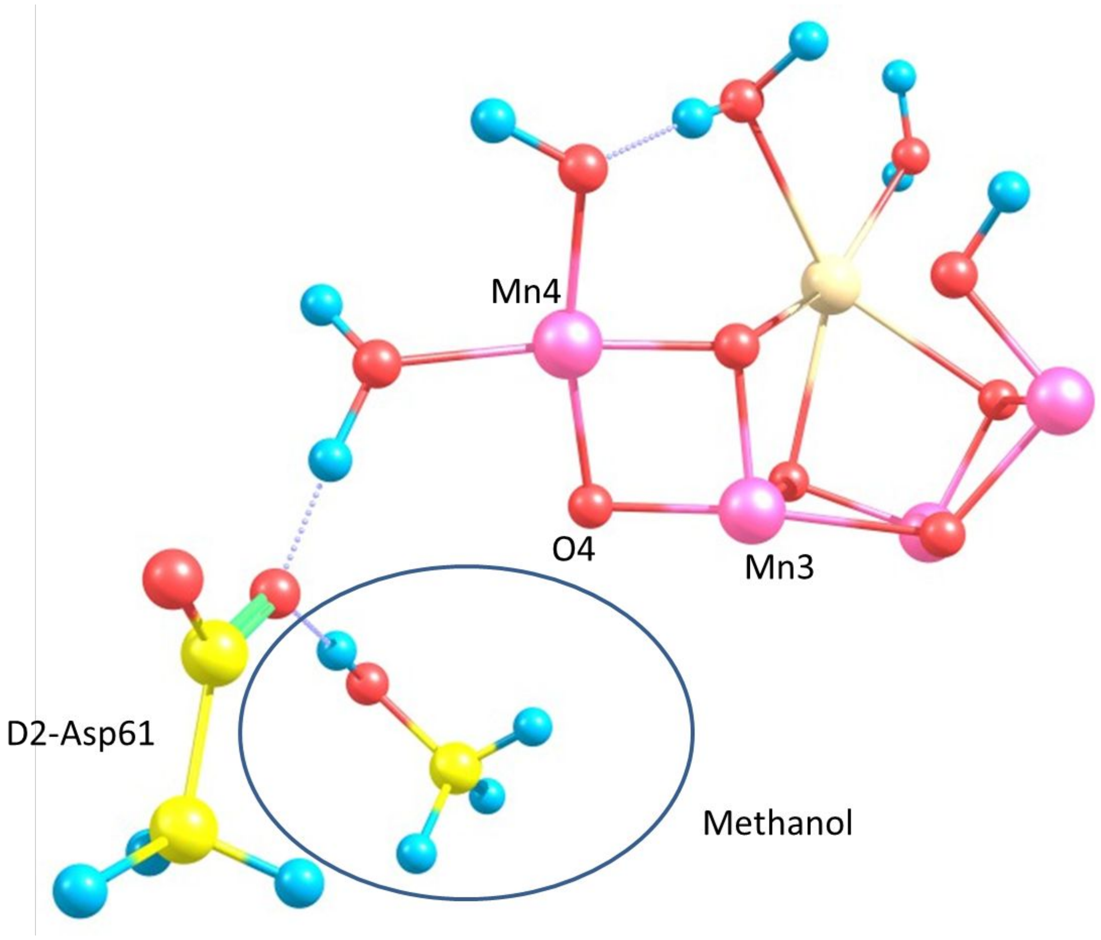

Figure S1. Selected atom illustration of models used with methanol molecule position highlighted. Oxygen (red), calcium (cream), manganese (pink), hydrogen (blue), carbon (yellow).

XYZ coordinates of methanol model used in BS-DFT calculations

Atom # (X, Y, Z)

20	-32.671515428	39.489307649	363.584180984
8	-33.018650467	39.748536667	366.012490981
8	-31.630716961	37.683797038	364.894248099
8	-33.693545758	37.372423491	366.352315303
8	-32.181899877	35.389270382	363.401140112
8	-33.681960113	37.265670638	363.336270384
8	-34.968544182	38.986973797	364.465081482
25	-34.711470325	38.985227367	366.239891309
25	-32.037498697	38.311959898	366.554614723
25	-32.943433739	36.353132769	364.703527054

25	-33.344217995	36.033588254	362.054055568
6	-32.875113052	30.227233866	362.898130926
6	-33.257622092	31.395042748	361.984135629
8	-32.360542496	32.318333874	361.820893494
8	-34.403908518	31.414474412	361.462621006
1	-33.524676000	29.356598865	362.717612791
1	-33.001077215	30.548678399	363.948751336
1	-31.816516813	29.949496653	362.760757831
6	-29.225257007	44.614365003	356.400966994
6	-30.366979934	43.570741299	356.453414923
6	-31.200535683	43.515225605	357.729782243
6	-31.345307452	42.316244349	358.463645015
6	-31.924030051	44.635829039	358.204329144
6	-32.161213305	42.236564841	359.607849748
6	-32.753733162	44.567090192	359.336042047
6	-32.891411942	43.358085590	360.073008419
8	-33.661837804	43.278519411	361.182751116
1	-29.610084731	45.642369200	356.530778952
1	-31.049564386	43.761533720	355.599951753
1	-29.942557370	42.564394144	356.275749254
1	-30.790926354	41.423826991	358.140498360
1	-31.855178995	45.589740670	357.663058338
1	-32.202948534	41.321349296	360.211407901
1	-33.311039606	45.457070502	359.653952008
1	-28.482024680	44.430485352	357.198486816
1	-28.705838368	44.568879467	355.427547392
6	-29.415390997	43.202243003	361.215080004
6	-29.747502929	43.996907487	362.459418143
7	-29.006949080	45.099795013	362.686811715
8	-30.683310240	43.677729660	363.233975593
1	-28.351776411	43.289235014	360.940075277
1	-30.025715370	43.579674113	360.375998117

1	-28.247781433	45.370655748	362.070677880
1	-29.197484503	45.681310098	363.498341907
1	-29.679672066	42.145440618	361.370445074
6	-30.219861971	34.365225051	357.883357042
6	-31.264096635	35.429758651	358.117025507
8	-32.463714493	35.104410694	358.331224329
1	-30.533794659	33.734293360	357.034150692
7	-30.842932860	36.704469168	358.093937871
6	-31.676155581	37.874163704	358.348179859
6	-31.171256001	38.702690021	359.529733928
6	-31.448563546	38.116708195	360.931109749
8	-31.157661934	38.817630615	361.932155843
8	-31.995525321	36.946578890	360.963755688
1	-29.858206586	36.867096973	357.897160585
1	-32.700187564	37.519071515	358.537416290
1	-30.089428435	38.919347455	359.448870324
1	-31.681096673	39.677518400	359.520819289
1	-30.173440456	33.715311787	358.775175001
1	-29.216559917	34.774704247	357.683936104
1	-31.697749301	38.504999662	357.441039969
6	-36.337962993	42.830694024	366.005324987
6	-36.059797980	41.456786143	365.501460922
8	-36.382664790	41.096982992	364.336549986
8	-35.486283189	40.709865476	366.390585045
1	-35.458822294	43.223743877	366.539444829
1	-37.155254966	42.774747880	366.744430984
1	-36.634765382	43.509462640	365.191024811
6	-36.143600990	48.444124976	363.039909998
6	-35.122114550	47.382151989	362.845162295
6	-35.195294116	46.136337470	362.251254139
7	-33.810233170	47.514661506	363.275664056
6	-33.117765802	46.401143274	362.961822892

7	-33.946011775	45.554174302	362.339571266
1	-37.078396458	48.162511210	362.529728867
1	-35.800678537	49.410399679	362.630148296
1	-36.031380813	45.629757502	361.769891181
1	-32.065125913	46.225377064	363.173997594
1	-33.737356214	44.562289678	361.873667286
1	-36.372220759	48.596705714	364.110385780
6	-34.808006004	51.803416027	365.723829003
6	-34.386315225	50.357829315	365.478854244
7	-34.895926059	49.420913707	366.309280743
8	-33.610922699	50.049181628	364.541982188
1	-35.459898836	51.915806322	366.604219076
1	-35.336695118	52.172592751	364.827861769
1	-34.643025271	48.437919081	366.195561538
1	-35.523881748	49.664747136	367.068097423
1	-33.902592135	52.420273496	365.855040043
1	-33.479173897	48.358787239	363.789000854
6	-40.803980999	40.323172986	369.348550007
6	-40.848031648	38.953172220	369.950417086
8	-40.347974614	37.940117922	369.394666397
1	-41.280108095	40.302989348	368.352350293
7	-41.456783957	38.852048652	371.139561562
6	-41.546156001	37.584363004	371.824189994
1	-41.862460764	39.679774197	371.564253446
1	-40.541096516	37.171595426	372.023130073
6	-39.840339010	36.699967996	365.631931016
6	-38.579261319	37.310607606	366.164441632
6	-37.307758181	37.464382483	365.628882112
7	-38.524716484	37.918373256	367.414035213
6	-37.288156326	38.415177111	367.613849183
7	-36.520954335	38.151712805	366.545529790
1	-39.664847758	36.285120625	364.626407918

1	-40.645213318	37.453601995	365.556438821
1	-36.907750300	37.164327652	364.660781940
1	-36.974032125	38.989547870	368.484615087
1	-40.202469845	35.884310743	366.283851205
1	-39.304359747	37.973430132	368.124317739
1	-42.104053585	36.845784259	371.221297832
1	-42.068466251	37.730258483	372.781413125
1	-39.751913744	40.628041535	369.198847670
1	-41.306485623	41.081147442	369.969988745
6	-33.839932002	34.207894009	371.940714991
6	-34.110036300	34.785743916	370.579052407
6	-33.429387144	35.705822200	369.793527194
7	-35.223046566	34.424750473	369.823164664
6	-35.229685904	35.075821344	368.646780495
7	-34.147533775	35.857865735	368.613937379
1	-34.669060307	34.432897830	372.634702379
1	-32.913026495	34.638464944	372.349982828
1	-32.501879135	36.254045511	369.982433181
1	-35.975492884	34.980551229	367.859703913
1	-33.922017317	36.469631555	367.779796862
1	-33.724029162	33.110962039	371.885105245
1	-35.937319863	33.758326098	370.110468572
6	-33.607831002	39.695785988	370.356862037
6	-33.539307658	39.180210233	368.941013757
8	-32.396028364	38.804355088	368.489548008
8	-34.607143989	39.163101228	368.252074871
1	-34.520297285	39.324248486	370.850788978
1	-33.684058067	40.798576333	370.301701842
1	-32.714668302	39.416295399	370.934635639
6	-29.063083976	41.225435884	366.547867925
6	-30.098917974	40.303691598	365.929960125
8	-30.565023634	40.475283327	364.786145699

8	-30.446664359	39.328978663	366.742123327
1	-28.564924691	41.829407102	365.773704568
1	-28.324925708	40.654210333	367.133444095
1	-29.597955878	41.901276893	367.240103039
6	-30.495675919	34.518567080	367.650801117
6	-31.300508816	35.565549423	366.971648039
8	-31.122100954	36.774874901	367.361713117
8	-32.087730569	35.190529804	366.052603530
1	-30.943028927	33.523104922	367.512510448
1	-30.377276994	34.735549239	368.724933160
1	-29.484438126	34.511253484	367.202843442
6	-25.861543109	37.415810044	363.979978037
7	-26.949903536	36.526300648	363.519965514
6	-28.278483867	36.687665220	363.713695198
7	-28.759135081	37.772549499	364.343819362
7	-29.151936942	35.751692074	363.302164394
1	-25.829465352	38.353652289	363.396201937
1	-25.986702795	37.652499072	365.050603203
1	-26.680215373	35.737278952	362.940643393
1	-28.203570117	38.618032169	364.399558058
1	-29.779088560	37.847849205	364.488897701
1	-28.883034091	34.808606303	363.017497328
1	-30.169667423	35.895959324	363.368092785
1	-24.914331300	36.875418174	363.844140027
1	-32.713836252	34.678933656	359.949638737
8	-30.509794567	37.544313818	370.131170906
1	-30.131817349	37.050552781	369.377265004
1	-31.122901773	38.154568017	369.654432576
8	-34.629465054	36.908356084	361.024693158
1	-34.798552724	36.470953184	360.148283154
8	-37.854228006	44.252038213	368.955000019
1	-37.618505958	43.275932436	369.064642000

1	-38.487902041	44.282437532	368.215682545
8	-33.052755251	34.508678728	360.870063280
1	-32.772650699	33.539989303	361.252166507
8	-35.781444934	41.520588180	361.611235404
1	-35.089166301	42.212458537	361.405134176
1	-36.015564273	41.617535838	362.564964207
8	-38.272544117	35.121211607	361.121131888
1	-37.992364355	35.889171298	360.556509779
1	-37.601062881	34.432600212	360.918671482
8	-35.888137391	33.539037510	360.569896840
1	-35.562184551	34.105576711	361.316666006
1	-35.331935388	32.719051302	360.728947406
8	-32.694642306	41.860651555	363.147945276
1	-31.788636183	42.279812444	363.178732435
1	-33.096283012	42.325570310	362.326635901
8	-35.239509013	35.449820359	358.717569291
1	-35.392293520	34.643022496	359.296992191
1	-34.302314144	35.386697790	358.417380292
8	-36.904060532	39.858062527	359.443779144
1	-35.997048386	39.940636531	359.037415665
1	-36.807047625	40.383269418	360.269083289
8	-35.284833441	45.211656821	368.691962422
1	-36.254675681	44.985183186	368.634474362
1	-34.993629086	45.497581733	367.788355580
8	-34.490826011	42.628521257	369.137899091
1	-33.839635531	42.386260971	368.413011453
1	-34.695423357	43.597007556	368.982289162
8	-32.561789964	42.087017254	367.300442382
1	-32.734801256	41.218341560	366.837924455
1	-32.579394467	42.752061163	366.565170065
8	-34.018332638	39.455421826	361.422703337
1	-34.792950428	40.075098154	361.543919547

1	-34.377432248	38.509734307	361.288218593
8	-34.018475646	46.096253773	366.327146594
1	-33.219869983	46.390627349	366.805376613
1	-33.685644825	45.327314999	365.791575241
8	-34.244727559	39.991399059	358.712087122
1	-33.864851135	40.882440098	358.569096321
1	-34.017498944	39.788899650	359.663965545
8	-32.779482789	43.847705671	365.121823544
1	-31.894222403	44.076043136	364.749252760
1	-33.066667003	43.145572157	364.476649460
8	-36.998442994	41.706679431	369.240833889
1	-36.011726263	41.914906983	369.124046100
1	-37.078759095	41.455446929	370.179077236
6	-36.376178960	33.719203039	364.230245990
6	-35.168862137	34.631317030	363.964511570
8	-34.535230661	35.138497460	364.950239122
8	-34.843492696	34.847956081	362.745045513
1	-37.241978016	34.102292456	363.662961269
1	-36.144438225	32.708469477	363.851810888
1	-36.600578775	33.679720652	365.305513994
8	-37.496030732	37.118464941	359.268286623
1	-37.249506482	38.065880679	359.448364859
1	-36.662164125	36.686346994	358.968749826
1	-35.653526521	39.709018666	364.258264548
6	-30.173037937	32.767165174	364.373334559
1	-29.238036138	32.982120702	364.920953973
1	-30.527302516	31.762165853	364.682176475
8	-29.884549821	32.822398864	362.968997534
1	-30.934707787	33.518043683	364.657134382
1	-30.758365174	32.668985702	362.514657781

XYZ coordinates of glycerol model used in BS-DFT calculations

Atom # (X, Y, Z)

20	-32.770637535	39.509747637	363.577017693
8	-33.047204609	39.748895257	366.011100861
8	-31.697160344	37.707544042	364.853960830
8	-33.706387630	37.358984262	366.340966834
8	-32.280485674	35.363644248	363.353947703
8	-33.740416035	37.270107787	363.329777028
8	-35.014845652	38.979773848	364.480263368
25	-34.733562071	38.978602015	366.251785809
25	-32.053833669	38.308796693	366.529027316
25	-32.980411458	36.354954879	364.684981259
25	-33.433387233	36.049890427	362.022703496
6	-32.875113307	30.227233879	362.898131289
6	-33.392742114	31.399521962	362.050088842
8	-32.539761981	32.352575159	361.866608924
8	-34.574283635	31.377110600	361.606195099
1	-33.557770127	29.364450859	362.847428301
1	-32.793317614	30.557593752	363.949943355
1	-31.865731132	29.931292616	362.565265117
6	-29.225256615	44.614364720	356.400966597
6	-30.368900098	43.571728969	356.448347736
6	-31.209493549	43.515404800	357.720301973
6	-31.333829752	42.324027409	358.469894132
6	-31.960558796	44.626452203	358.174657002
6	-32.155227930	42.241951369	359.610461441
6	-32.795920686	44.555020859	359.302003544
6	-32.912021861	43.354019027	360.055891408
8	-33.687343056	43.276115141	361.162681234
1	-29.609790117	45.642501245	356.530968294
1	-31.047029254	43.763553401	355.591707244

1	-29.944127718	42.565217864	356.272420248
1	-30.758828362	41.439186862	358.162214516
1	-31.909483019	45.573779310	357.619918278
1	-32.178821364	41.333787052	360.225921746
1	-33.375382806	45.436442483	359.604103795
1	-28.485008756	44.428554712	357.200793474
1	-28.702752651	44.569221732	355.429207503
6	-29.415390682	43.202242844	361.215079876
6	-29.743187709	43.975977603	362.473892445
7	-28.966066536	45.042691723	362.747197860
8	-30.707425678	43.673068719	363.219440268
1	-28.353356419	43.292344736	360.935704722
1	-30.031621066	43.592230465	360.386307786
1	-28.186061640	45.302566795	362.152785526
1	-29.155003193	45.611346170	363.568195361
1	-29.680405979	42.143206746	361.354453368
6	-30.219862172	34.365225156	357.883356707
6	-31.265979343	35.428872538	358.117838683
8	-32.465267696	35.105788785	358.330534722
1	-30.548964550	33.711636798	357.057675238
7	-30.839285165	36.702884313	358.095611476
6	-31.665713258	37.878695414	358.340148099
6	-31.171256296	38.702689534	359.529733563
6	-31.510990334	38.137073983	360.926059524
8	-31.260104607	38.857290173	361.925789446
8	-32.059310129	36.967852244	360.956250203
1	-29.853368899	36.859965935	357.900835845
1	-32.696000942	37.533213534	358.513109531
1	-30.080474517	38.881191404	359.482179370
1	-31.646576485	39.694196408	359.496617282
1	-30.147770517	33.740422289	358.791633663
1	-29.224445605	34.776986685	357.651665274

1	-31.667502676	38.510667898	357.433455709
6	-36.337962692	42.830693795	366.005324996
6	-36.082419250	41.449748795	365.510459304
8	-36.413263777	41.089277359	364.347984554
8	-35.515433623	40.699886335	366.402668576
1	-35.443968741	43.220313110	366.517256166
1	-37.140162322	42.792452540	366.761789781
1	-36.643302232	43.503811115	365.189557026
6	-36.143600871	48.444125326	363.039910483
6	-35.123609713	47.382132026	362.838301942
6	-35.199653810	46.139209793	362.238802590
7	-33.810679734	47.511031739	363.266643682
6	-33.119951259	46.398220910	362.946022278
7	-33.950835523	45.555350590	362.321704350
1	-37.079138753	48.166431264	362.528939229
1	-35.799770232	49.412176143	362.635238065
1	-36.037461143	45.635810860	361.757156956
1	-32.067032853	46.219942580	363.154855679
1	-33.748024271	44.564223397	361.855206387
1	-36.371319270	48.591177503	364.111367615
6	-34.808006280	51.803415642	365.723829254
6	-34.389116229	50.356567663	365.478031419
7	-34.900413817	49.420742363	366.308155680
8	-33.614215855	50.046695296	364.541090258
1	-35.462549617	51.916104440	366.602205658
1	-35.332490870	52.175503709	364.826666280
1	-34.649320141	48.437188906	366.194416085
1	-35.527725818	49.665950078	367.067050263
1	-33.901318725	52.417481303	365.859278837
1	-33.477598494	48.353140369	363.781485225
6	-40.803980832	40.323172631	369.348550475
6	-40.855968218	38.952780719	369.949004348

8	-40.369567952	37.935565434	369.388364156
1	-41.283809038	40.307768986	368.354114958
7	-41.455288319	38.853803668	371.142828740
6	-41.546155973	37.584363353	371.824189927
1	-41.851109818	39.683199214	371.573574078
1	-40.541845533	37.164412927	372.011301415
6	-39.840339363	36.699967859	365.631930627
6	-38.583577802	37.302559191	366.176402222
6	-37.310655256	37.454143008	365.646538545
7	-38.533556785	37.903328339	367.429208074
6	-37.296992563	38.395925819	367.636599636
7	-36.527170554	38.135167962	366.569529791
1	-39.660204599	36.295181373	364.623092100
1	-40.644150784	37.455117738	365.559490691
1	-36.907844564	37.159698803	364.677995025
1	-36.984073510	38.965473619	368.510817782
1	-40.208132157	35.878040518	366.272838325
1	-39.318639243	37.960609973	368.134409644
1	-42.115088222	36.851597914	371.224587200
1	-42.057684083	37.730554472	372.787184535
1	-39.750136498	40.620426693	369.195685252
1	-41.298460772	41.083933449	369.973009825
6	-33.839931995	34.207894354	371.940715029
6	-34.108027329	34.777232952	370.575421582
6	-33.429667415	35.698305753	369.789369112
7	-35.215159910	34.405363104	369.816088401
6	-35.221339978	35.052012948	368.637402372
7	-34.143546603	35.840480485	368.606111833
1	-34.673410397	34.430788862	372.630182504
1	-32.917629242	34.646522708	372.351819779
1	-32.504758179	36.251183341	369.979311743
1	-35.963843410	34.949842959	367.848076467

1	-33.921423054	36.453986184	367.773590115
1	-33.716490487	33.111487280	371.891177057
1	-35.925936257	33.734937087	370.102778848
6	-33.607831281	39.695786438	370.356861884
6	-33.542164489	39.171972493	368.940956865
8	-32.404281155	38.791190651	368.480411875
8	-34.615365625	39.153429287	368.258805846
1	-34.507766511	39.306825070	370.860268505
1	-33.707953370	40.796258759	370.295364291
1	-32.703624804	39.437948636	370.927449455
6	-29.063084358	41.225436123	366.547868088
6	-30.109933178	40.310921559	365.930041933
8	-30.569000837	40.488212088	364.778710271
8	-30.468724690	39.339930992	366.732109671
1	-28.560326114	41.822864197	365.771641873
1	-28.331602699	40.645650701	367.133343812
1	-29.589391329	41.908414548	367.239524018
6	-30.495676522	34.518567241	367.650800958
6	-31.281300687	35.559614846	366.929528614
8	-31.123682362	36.772521195	367.320442213
8	-32.041472150	35.180302251	365.987633007
1	-30.917104313	33.517663213	367.475575443
1	-30.465225125	34.728886622	368.732609031
1	-29.450806653	34.522104449	367.288078669
6	-25.861543029	37.415810191	363.979978216
7	-27.149398360	36.788494260	363.728870459
6	-28.306655645	37.467082447	363.662734630
7	-28.317205760	38.807767315	363.824901514
7	-29.439212803	36.823411507	363.386558375
1	-25.667447238	38.225319118	363.253727658
1	-25.789185351	37.828146699	365.005191548
1	-27.235286134	35.763014036	363.794956231

1	-27.549870926	39.263715721	364.306227200
1	-29.207414534	39.308732911	363.805926212
1	-29.451728318	35.845102750	363.049315153
1	-30.336173726	37.250743551	363.640847547
1	-25.076212966	36.654526621	363.861374422
1	-32.745005420	34.678419669	359.981438954
8	-30.534916207	37.515808543	370.124966058
1	-30.121144203	37.024106929	369.389218715
1	-31.132528738	38.123794577	369.625432414
8	-34.704947965	36.937070291	360.994615895
1	-34.860933182	36.490703324	360.120305271
8	-37.868885442	44.250315927	368.955394751
1	-37.633609370	43.274579741	369.068804878
1	-38.509999194	44.277179356	368.222366045
8	-33.149188836	34.512236362	360.873022927
1	-32.904260070	33.495527328	361.300982871
8	-35.846458200	41.554453295	361.597235900
1	-35.141172100	42.233054440	361.392967785
1	-36.073046605	41.647404048	362.552659144
8	-38.342361394	35.152193534	361.022903294
1	-38.041555548	35.906679254	360.451184673
1	-37.664653513	34.457492416	360.865068480
8	-35.968090306	33.520703763	360.588523373
1	-35.639467922	34.111323661	361.313588596
1	-35.448654498	32.687691511	360.804999176
8	-32.737562643	41.876932332	363.141518495
1	-31.826364315	42.284819811	363.167521636
1	-33.132760185	42.338093408	362.312499251
8	-35.254869402	35.417927975	358.704598800
1	-35.415079874	34.620023139	359.290417317
1	-34.308555784	35.358360523	358.432136655
8	-36.911027360	39.860623191	359.378365106

1	-35.991647143	39.957252960	359.003286702
1	-36.850906379	40.376539927	360.211779528
8	-35.301249264	45.211125462	368.688171579
1	-36.270299491	44.982574889	368.626983110
1	-35.007408333	45.498119224	367.785845697
8	-34.503881643	42.629919340	369.139058907
1	-33.856084708	42.388780078	368.410902274
1	-34.711318663	43.597805254	368.982466542
8	-32.585039503	42.090250638	367.289916961
1	-32.757604925	41.219607512	366.830861888
1	-32.604715294	42.753917799	366.553162129
8	-34.103975460	39.477854412	361.431298787
1	-34.878810936	40.100706642	361.534071857
1	-34.461657832	38.535281899	361.278855792
8	-34.032221678	46.105598546	366.326742924
1	-33.235315213	46.404095275	366.805369133
1	-33.695180887	45.340334608	365.788677784
8	-34.235616915	40.011325134	358.719003356
1	-33.845114490	40.898777224	358.580898344
1	-34.033592620	39.812089670	359.677135599
8	-32.796503633	43.856707085	365.116061934
1	-31.908789215	44.077931546	364.744680718
1	-33.090763825	43.161359697	364.466470104
8	-37.011940792	41.705962031	369.248564876
1	-36.025575912	41.914255287	369.131398252
1	-37.092282500	41.458117391	370.187700902
6	-36.376178862	33.719202696	364.230245656
6	-35.199539619	34.650174829	363.956323164
8	-34.546270291	35.135039930	364.940175141
8	-34.922106528	34.899665440	362.731899889
1	-37.259672117	34.079838657	363.676341456
1	-36.125817041	32.715040632	363.844660599

1	-36.588134617	33.666768497	365.307612404
8	-37.512010761	37.119249312	359.156511410
1	-37.258838162	38.061331691	359.352523208
1	-36.673700106	36.674670701	358.889618129
1	-35.705296758	39.698892244	364.276784529
6	-29.746434393	33.117249729	363.179003828
1	-29.169745857	32.426749834	362.529870883
1	-30.676803718	32.590521991	363.464329027
6	-28.918517916	33.445683725	364.419231450
1	-29.409963955	34.265543740	364.974804773
6	-28.729570182	32.246375947	365.350184305
1	-29.703799603	31.933057126	365.766171288
1	-28.314994583	31.389349229	364.774872599
8	-30.060782335	34.282841118	362.414137325
1	-30.961239155	34.604542618	362.728091479
8	-27.881064253	32.576095950	366.456891948
1	-27.120447299	33.055066018	366.067800585
8	-27.608812862	33.979827660	364.069939520
1	-27.219197292	33.395917970	363.388560631

S2 ELDOR-detected NMR (EDNMR) simulations

The original *horseradish* code was modified slightly to run the algorithm using the developmental version of *Easyspin* available at Github repository: https://github.com/StollLab/EasySpin

All MATLAB code used for the ELDOR-detected NMR simulations in this work can be found in the GitHub repository:

https://github.com/ciaranrogers16/MATLAB-scripts-for-EPR/tree/main/The-magnetic-and-electronic-structure-properties-of-the-S3-state-of-Nature%E2%80%99s-water-oxidising-complex:-a-combined-Eldor-Detected-Nuclear-Magnetic-Resonance-spectral-simulation-and-Broken-Symmetry-Density-Functional-Theory-study.

Supplementary EDNMR simulations

Simulation of Mn^{IV} centres (as assigned in main text – Fig 3) before summation, plotted relative to experimental EDNMR spectra, with an applied Lorentzian (FWHM = 400 MHz) function. The proton peak is associated with each of the Mn ions in the simulation. Resultantly all four Mn ions contribute to this peak around the proton Larmor frequency. This replicates the intensity of the spectral feature seen in the experimental data for the mononuclear calculations in the main text.

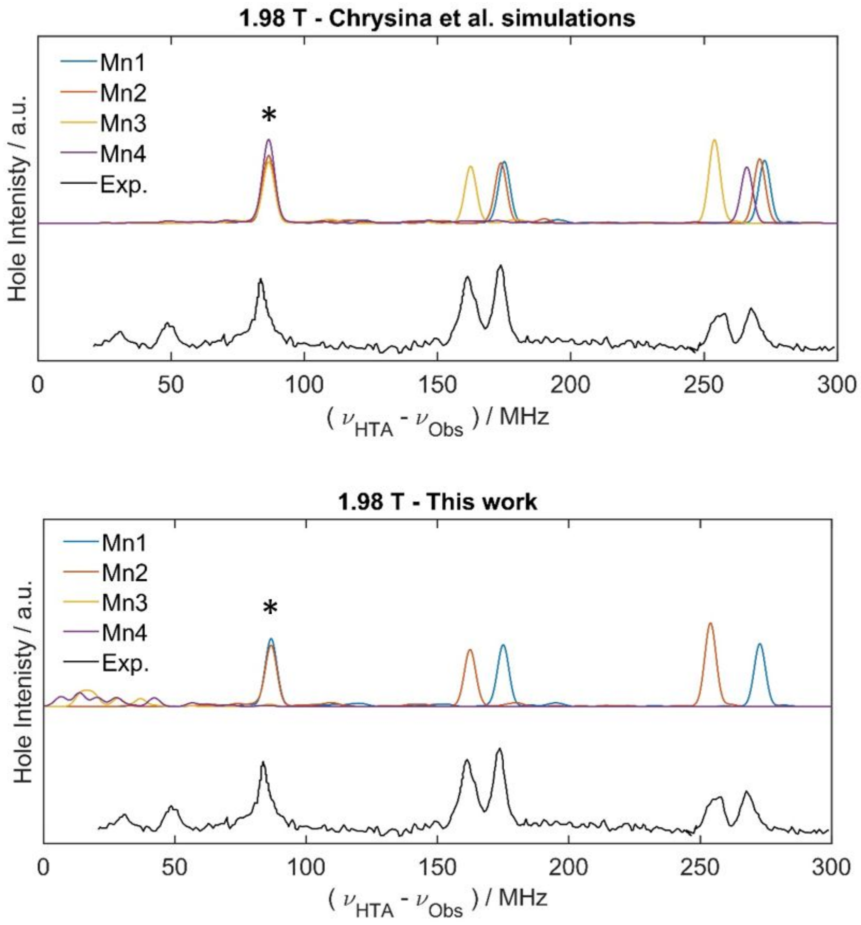

Figure S2: Spectral simulations using *horseradish* algorithm showing individual mononuclear Mn hyperfine coupling contributions compared to experimental spectrum (black) at 1.98 T. ^{1}H peak denoted with an asterisk. Experimental EDNMR spectra (black)reproduced from reference 25.

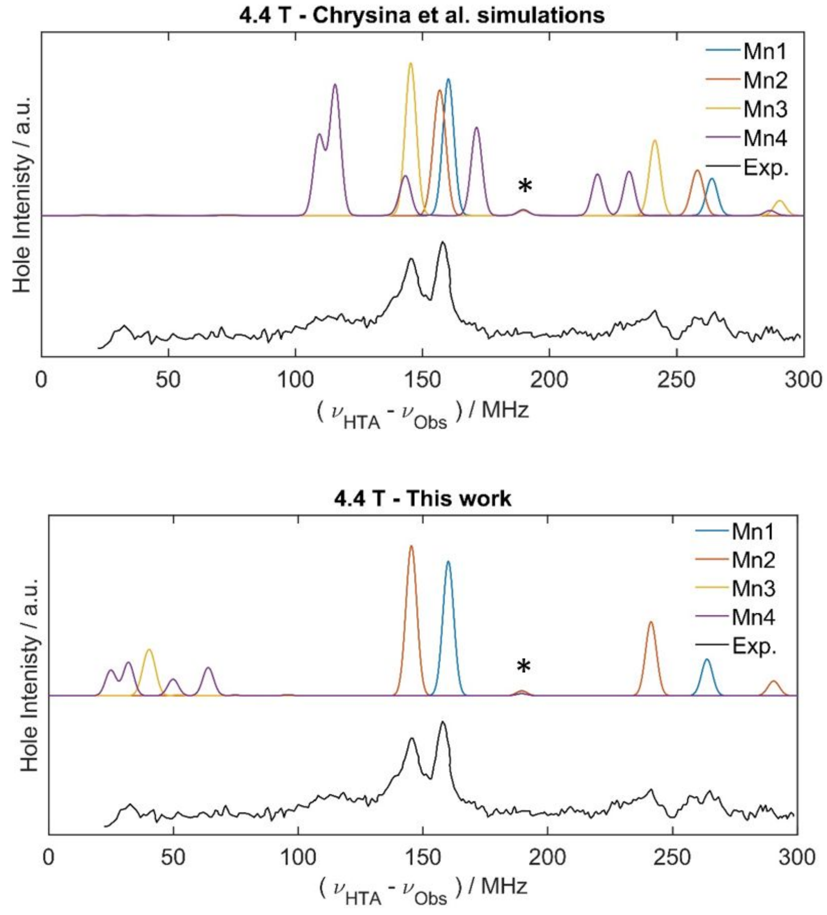

Figure S3: Spectral simulations using *horseradish* algorithm showing individual mononuclear Mn hyperfine coupling contributions compared to experimental spectrum (black) at 4.4 T. ^{1}H peak denoted with an asterisk. Experimental EDNMR spectra (black) reproduced from reference 25.

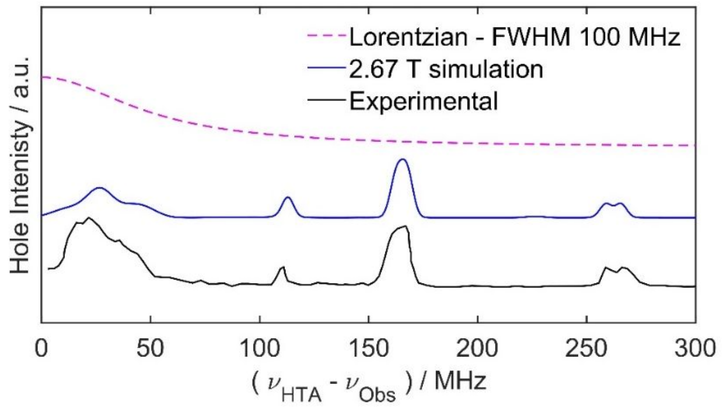

Figure S4: Spectral simulations using *horseradish* algorithm for the native Ca form of S_3 state of the WOC (blue) compared to experimental spectrum (black)[25] at 2.67 T, with a Lorentzian bandwidth of FWHM = 100 MHz). Experimental EDNMR spectra (black) reproduced from reference 25.

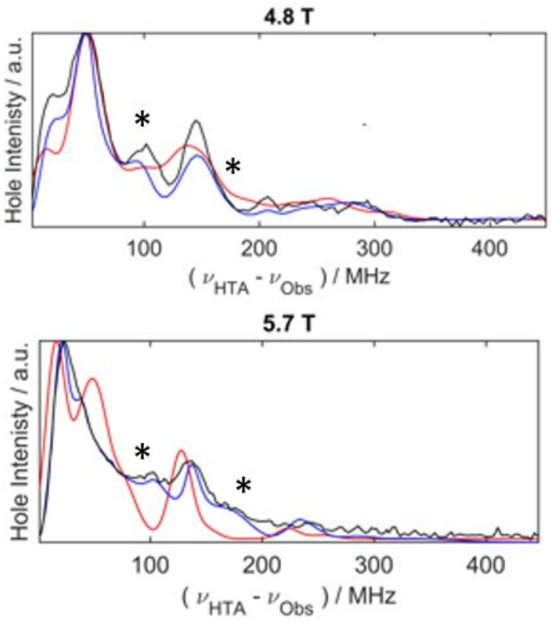

Figure S5: Spectral simulations using the *horseradish* algorithm for the MeOH trapped form of the S_3 state of the WOC (red) compared to experimental spectrum (black) recorded by Marchiori *et al.*[26] and original simulations using *salt* (blue) at D-band (~130 GHz), with a Lorentzian bandwidth of FWHM = 300 MHz. The small ^{55}Mn couplings occur in a region overlapping with ^{14}N resonances. Small deviations of the simulations occur at resonance frequencies where multi-quantum transitions are expected to be present, as these are not included in the *horseradish* simulations (red) shown, which may further affect the amplitude and shift the position of the single quantum transitions. Marchiori *et al.* have included MQTs in their simulations,[26] with the asterisks showing where MQTs are expected to occur in the spectrum.

Bandwidth considerations in simulating EDNMR spectra

As discussed in the main text, in order to model the effects of the bandwidth in the simulations of the experimental EDNMR data, a Lorentzian function with a FWHM of 400 MHz was chosen, as this best reproduced the results of the experimental spectra, in particular the relative intensities of the spectral features at both low and high fields. The simulation further assumes a symmetric bandwidth about the detection frequency (ν_{Obs}), as experimental spectra are often collected either side of ν_{Obs} before summation of both sides to give the final spectrum. A symmetric bandwidth however cannot be assumed, and an asymmetry of this bandwidth may give rise to further inhomogeneities of the B_1 field. The effect of varying the line-shape and FWHM of the applied simulated bandwidth is shown below.

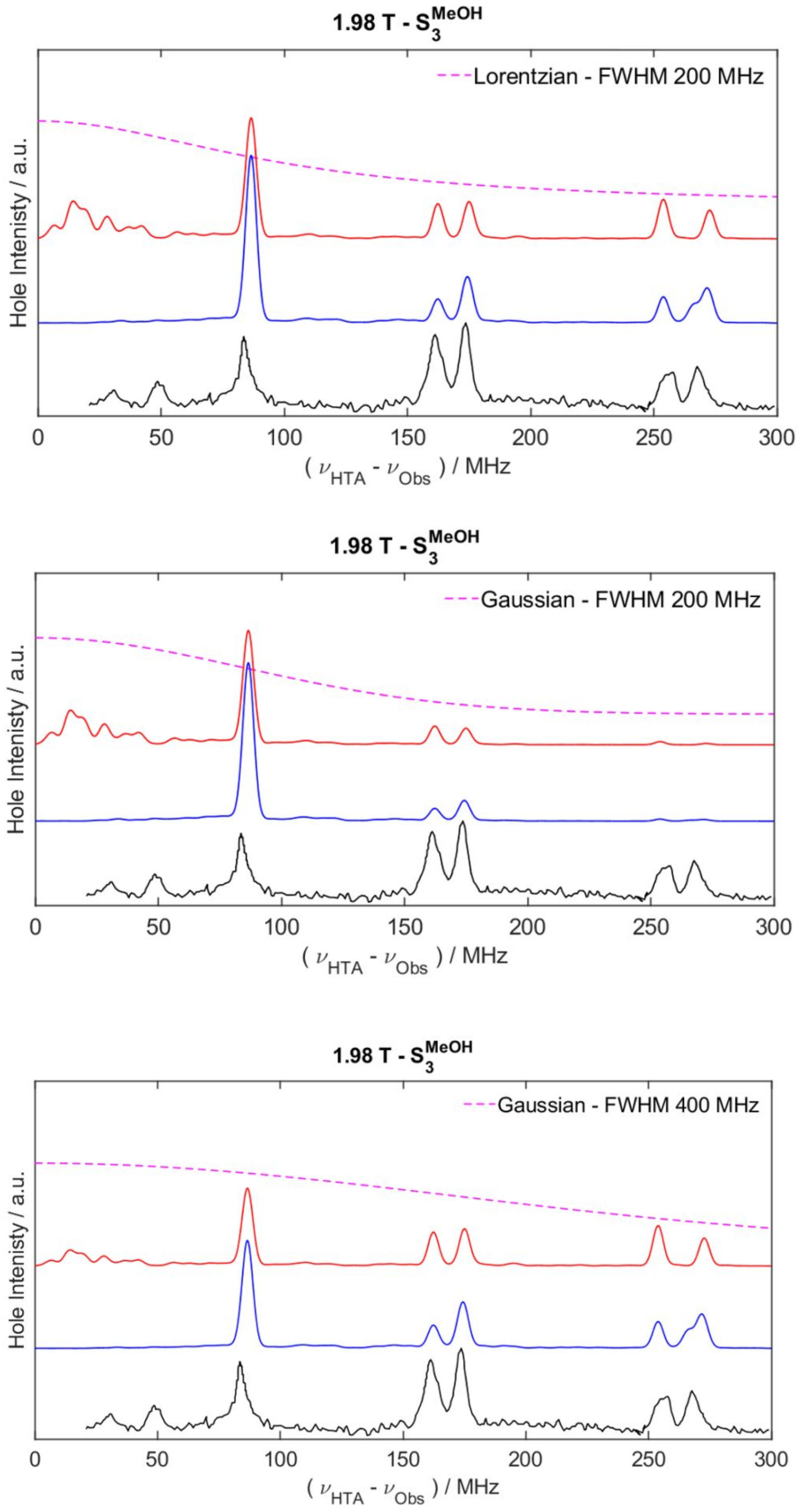

Figure S6: Variation in the applied bandwidth (Lineshape and FWHM) in simulations (red – this work; blue – Chrysina *et al.* simulation[25]) of experimental EDNMR data (black) on the MeOH trapped S_3 state in the WOC of PSII at 1.98 T.

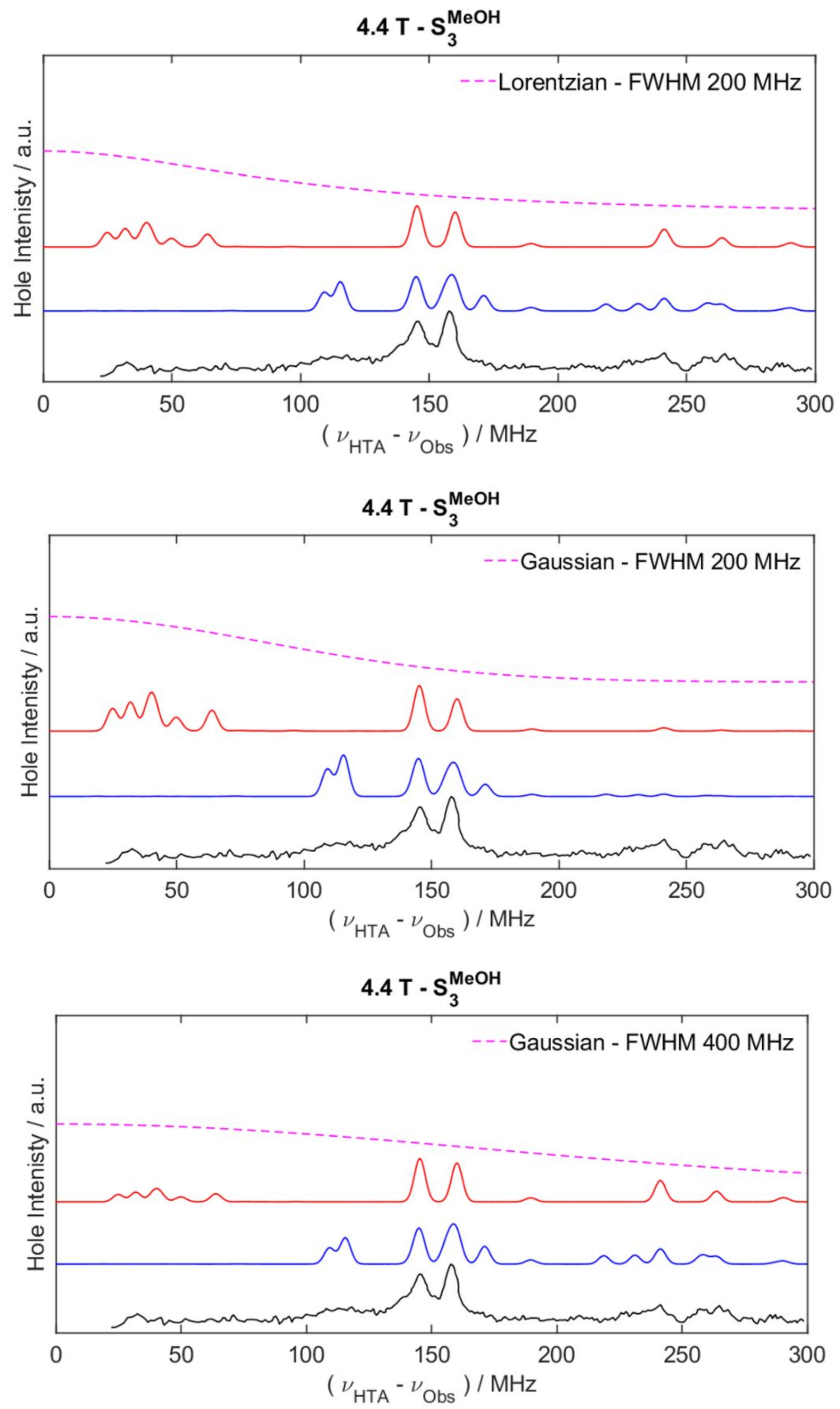

Figure S7: Variation in the applied bandwidth (Lineshape and FWHM) in simulations (red – this work; blue – Chrysina *et al.* simulation[25]) of experimental EDNMR data (black) on the MeOH trapped S_3 state in the WOC of PSII at 4.4 T.

Consideration of multi-quantum effects in EDNMR

EDNMR simulations were performed including the coupling to two of the four Mn ions in turn and a single proton (it was not possible to include the couplings to all four Mn ions in the same simulation due to computational restraints). Four simulations including couplings to [Mn_1, Mn_2, ^1H], [Mn_1, Mn_4, ^1H], [Mn_2, Mn_4, ^1H] and [Mn_3, Mn_4, ^1H], were performed and the results summed. The simulations were performed with different amplitudes of the HTA pulse of 4.7 x 10^7 rad s^{-1}, the same power used to generate the simulations shown in Figure 3 of the main paper, and 4.7 x 10^6 rad s^{-1} for both open and closed cubane models. The results are displayed with the experimental results (black trace) and shown in the main text (Figure 4). Results were calculated for both the 1.98 T and 4.4 T datasets. It can be seen by comparing the results between Figure 3 and Figure 4 that at higher HTA amplitudes the multinuclear spectra differ significantly from the mononuclear and the experimental spectrum. Chrysina *et al.*[25] report the amplitude of the HTA to be 4.7 x 10^7 rad s^{-1} at the centre of the blind spot however this will be lower further away from the central blind spot due to the resonator bandwidth. As such without an accurate report of the resonator bandwidth to include in the EDNMR calculation it is not possible to accurately determine the correct pulse amplitude that should be used at different frequency offsets. To this end as the pulse amplitude in the region $\upsilon_{HTA}-\upsilon_{Obs} > 140$ MHz, where most of the signals are observed will be lower than 4.7 x 10^7 rad s^{-1} and in this region the simulations for HTA pulse amplitude of 4.7 x 10^6 rad s^{-1} gives good agreement with the summed mononuclear simulations, it was determined that summation of the mononuclear simulations is a reasonable approximation in regions of the spectra where the HTA amplitude is below 4.7 x 10^7 rad s^{-1}. However, it must be remembered that without a recorded experimental resonator bandwidth it is not possible to know what the HTA amplitude should be at each offset frequency and consequently deviations in the calculated spectra presented in the main paper and the experimentally recorded data in the lower frequency region may be a result of intramolecular multi quantum transitions (MQTs) that are not considered in the simulations presented in Figure 3 of the main text. . Interestingly in the simulations of the 1.98 T data using a HTA pulse amplitude of 4.7 x 10^6 rad s^{-1}, the proton signal at 85 MHz offset is not reproduced (Figure 4, upper left plot) suggesting that the HTA pulse amplitude at this offset is likely greater than 4.7 x 10^6 rad s^{-1}, as it is present in the calculations with higher HTA pulse amplitude. As signals are seen at this offset due to MQTs in the 4.4T simulations using the closed cubane parameters with HTA pulse amplitudes of both 4.7 x 10^6 rad s^{-1} and 4.7 x 10^7 rad s^{-1}, Figure 4 (right, top and bottom (blue traces)), this may be an indication that these parameters do no accurately reproduce the experimental data in all regards, as it would be expected that while the exact HTA pulse amplitude at each offset is unknown that the HTA pulse power at a specific offset would be constant between data recorded at different field positions if only the field position is changed. Further the application of a Lorentzian function, as discussed above, causes an increase in the amplitude of the signals calculated at low frequency relative to those calculated at high frequency and

may artificially inflate the amplitude of the low frequency signals. In all the EDNMR simulations calculated with the parameters for an open cubane structure (red traces), all significant low frequency signals lie below 50 MHz offset frequency in a region where disentangling these from the central blind-spot can cause uncertainty.

References

(1) Neese, F. Software Update: The ORCA Program System, Version 4.0. *Wiley Interdiscip. Rev. Comput. Mol. Sci.,* 2018, **8**, 1, e1327.

(2) Corry, T. A.; O'Malley, P. J. Proton Isomers Rationalize the High- and Low-Spin Forms of the S_2 State Intermediate in the Water-Oxidizing Reaction of Photosystem II. *J. Phys. Chem. Lett.,* 2019, **10**, 17, 5226–5230.

(3) Becke, A. D. Density-Functional Exchange-Energy Approximation with Correct Asymptotic Behavior. *Phys. Rev. A,* 1988, **38**, 6, 3098–3100.

(4) Perdew, J. Density-Functional Approximation for the Correlation Energy of the Inhomogeneous Electron Gas. *Phys. Rev. B,* 1986, **33**, 12, 8822–8824.

(5) Lenthe, E. Van; Baerends, E. J.; Snijders, J. G. Relativistic Regular Two-Component Hamiltonians. *J. Chem. Phys.* 1993, **99**, 6, 4597–4610.

(6) van Lenthe, E.; Baerends, E. J.; Snijders, J. G. Relativistic Total Energy Using Regular Approximations. *J. Chem. Phys.* 1994, **101**, 11, 9783–9792.

(7) van Wüllen, C. Molecular Density Functional Calculations in the Regular Relativistic Approximation: Method, Application to Coinage Metal Diatomics, Hydrides, Fluorides and Chlorides, and Comparison with First-Order Relativistic Calculations. *J. Chem. Phys.* 1998, **109**, 2, 392-399.

(8) Weigend, F.; Ahlrichs, R. Balanced Basis Sets of Split Valence, Triple Zeta Valence and Quadruple Zeta Valence Quality for H to Rn: Design and Assessment of Accuracy. *Phys. Chem. Chem. Phys.* 2005, **7**, 18, 3297-3305.

(9) Eichkorn, K.; Treutler, O.; Öhm, H.; Häser, M.; Ahlrichs, R. Auxiliary Basis Sets to Approximate Coulomb Potentials. *Chem. Phys. Lett.* 1995, **240**, 4, 283–289.

(10) Eichkorn, K.; Weigend, F.; Treutler, O.; Ahlrichs, R. Auxiliary Basis Sets for Main Row Atoms and Transition Metals and Their Use to Approximate Coulomb Potentials. *Theor. Chem. Acc.* 1997, **97**, 119–124.

(11) Weigend, F. Accurate Coulomb-Fitting Basis Sets for H to Rn. *Phys. Chem. Chem. Phys.* 2006, **8,** 9, 1057–1065.

(12) Grimme, S.; Antony, J.; Ehrlich, S.; Krieg, H. A Consistent and Accurate *Ab Initio* Parametrization of Density Functional Dispersion Correction (DFT-D) for the 94 Elements H-Pu. *J. Chem. Phys.* 2010, **132**, 15, 154104.

(13) Grimme, S.; Ehrlich, S.; Goerigk, L. Effect of the Damping Function in Dispersion Corrected Density Functional Theory. *J. Comput. Chem.* 2011, **32**, 7, 1456–1465.

(14) Pantazis, D. A.; Ames, W.; Cox, N.; Lubitz, W.; Neese, F. Two Interconvertible Structures That Explain the Spectroscopic Properties of the Oxygen-Evolving Complex of Photosystem II in the S_2 State. *Angew. Chemie Int. Ed.* 2012, **51**, 39, 9935–9940.

(15) Cox, N.; Retegan, M.; Neese, F.; Pantazis, D. A.; Boussac, A.; Lubitz, W. Photosynthesis. Electronic Structure of the Oxygen-Evolving Complex in Photosystem II Prior to O-O Bond Formation. *Science* 2014, **345**, 6198, 804–808.

(16) Pantazis, D. A.; Orio, M.; Petrenko, T.; Zanten, V.; Bill, E.; Lubitz, W.; Messinger, J.; Neese, F. A New Quantum Chemical Approach to the Magnetic Properties of Oligonuclear Transition-Metal Complexes: Application to a Model for the Tetranuclear Manganese Cluster of Photosystem II. *Chem. - A Eur. J.* 2009, **15**, 20, 5108–5123.

(17) Staroverov, V. N.; Scuseria, G. E.; Tao, J.; Perdew, J. P. Comparative Assessment of a New Nonempirical Density Functional: Molecules and Hydrogen-Bonded Complexes. *J. Chem. Phys.* 2003, **119**, 23.

(18) Neese, F.; Wennmohs, F.; Hansen, A.; Becker, U. Efficient, Approximate and Parallel Hartree-Fock and Hybrid DFT Calculations. A "chain-of-Spheres" Algorithm for the Hartree-Fock Exchange. *Chem. Phys.* 2009, **356**, 98–109.

(19) Neese, F. The ORCA Program System. *Wiley Interdiscip. Rev. Comput. Mol. Sci.* 2012, **2**, 1, 73–78.

(20) Cox, N.; Ames, W.; Epel, B.; Kulik, L. V.; Rapatskiy, L.; Neese, F.; Messinger, J.; Wieghardt, K.; Lubitz, W. Electronic Structure of a Weakly Antiferromagnetically Coupled Mn(II) Mn(III) Model Relevant to Manganese Proteins: A Combined EPR, ^{55}Mn-ENDOR, and DFT Study. *Inorg. Chem.* 2011, **50**, 17, 8238–8251.

(21) Sinnecker, S.; Neese, F.; Noodleman, L.; Lubitz, W. Calculating the Electron Paramagnetic Resonance Parameters of Exchange Coupled Transition Metal Complexes Using Broken Symmetry Density Functional Theory: Application to a Mn(III)/Mn(IV) Model Compound. *J. Am. Chem. Soc.* 2004, **126**, 8, 2613–2622.

(22) Schinzel, S.; Kaupp, M. Validation of Broken-Symmetry Density Functional Methods for the Calculation of Electron Paramagnetic Resonance Parameters of Dinuclear

Mixed-Valence Mn(IV) Mn(III) Complexes. *Can. J. Chem.* 2009, **87**, 10, 1521–1539.

(23) Ames, W.; Pantazis, D. A.; Krewald, V.; Cox, N.; Messinger, J.; Lubitz, W.; Neese, F. Theoretical Evaluation of Structural Models of the S_2 State in the Oxygen Evolving Complex of Photosystem II: Protonation States and Magnetic Interactions. *J. Am. Chem. Soc.* 2011, **133**, 49, 19743–19757.

(24) Corry, T. A.; O'Malley, P. J. S3 State Models of Nature's Water Oxidizing Complex: Analysis of Bonding and Magnetic Exchange Pathways, Assessment of Experimental Electron Paramagnetic Resonance Data, and Implications for the Water Oxidation Mechanism. *J. Phys. Chem. B* 2021, **125**, 36, 10097–10107.

(25) Chrysina, M.; Heyno, E.; Kutin, Y.; Reus, M.; Nilsson, H.; Nowaczyk, M. M.; DeBeer, S.; Neese, F.; Messinger, J.; Lubitz, W.; Cox, N. Five-Coordinate Mn^{IV} Intermediate in the Activation of Nature's Water Splitting Cofactor. *Proc. Natl. Acad. Sci.* 2019, **116**, 34, 16841–16846.

(26) Marchiori, D. A.; Debus, R. J.; Britt, R. D. Pulse EPR Spectroscopic Characterization of the S3 State of the Oxygen-Evolving Complex of Photosystem II Isolated from Synechocystis. *Biochemistry* 2020, **59**, 51, 4864–4872.
